# *3-ketodihydrosphingosine reductase* mutation induces steatosis and hepatic injury in zebrafish

**DOI:** 10.1038/s41598-018-37946-0

**Published:** 2019-02-04

**Authors:** Ki-Hoon Park, Zhi-wei Ye, Jie Zhang, Samar M. Hammad, Danyelle M. Townsend, Don C. Rockey, Seok-Hyung Kim

**Affiliations:** 1 Department of Medicine, Medical University of South Carolina, Charleston, SC 29425 USA; 2 Department of Cell and Molecular Pharmacology and Experimental Therapeutics, Medical University of South Carolina, Charleston, SC 29425 USA; 3 Department of Regenerative Medicine and Cell Biology, Medical University of South Carolina, Charleston, SC 29425 USA

## Abstract

3-ketodihydrosphingosine reductase (KDSR) is the key enzyme in the *de novo* sphingolipid synthesis. We identified a novel missense *kdsr*^*I105R*^ mutation in zebrafish that led to a loss of function, and resulted in progression of hepatomegaly to steatosis, then hepatic injury phenotype. Lipidomics analysis of the *kdsr*^*I105R*^ mutant revealed compensatory activation of the sphingolipid salvage pathway, resulting in significant accumulation of sphingolipids including ceramides, sphingosine and sphingosine 1-phosphate (S1P). Ultrastructural analysis revealed swollen mitochondria with cristae damage in the *kdsr*^*I105R*^ mutant hepatocytes, which can be a cause of hepatic injury in the mutant. We found elevated *sphingosine kinase* 2 (*sphk2*) expression in the *kdsr*^*I105R*^ mutant. Genetic interaction analysis with the *kdsr*^*I105R*^ and the *sphk2*^*wc1*^ mutants showed that sphk2 depletion suppressed liver defects observed in the *kdsr*^*I105R*^ mutant, suggesting that liver defects were mediated by S1P accumulation. Further, both oxidative stress and ER stress were completely suppressed by deletion of *sphk2* in *kdsr*^*I105R*^ mutants, linking these two processes mechanistically to hepatic injury in the *kdsr*^*I105R*^ mutants. Importantly, we found that the heterozygous mutation in *kdsr* induced predisposed liver injury in adult zebrafish. These data point to kdsr as a novel genetic risk factor for hepatic injury.

## Introduction

Sphingolipids are essential lipid components of eukaryotic cell membranes and play important roles in membrane trafficking, cell proliferation, differentiation, apoptosis, and cell migration^1,2^. Sphingolipids are synthesized via a *de novo* synthetic pathway as well as a salvage pathway. The *de novo* synthesis of sphingolipids begins with condensation of the palmitoyl-CoA and L-serine by serine palmitoyltransferase, to produce 3-ketodihydrosphingosine. 3-ketodihydrosphingosine is then reduced by KDSR to generate dihydrosphingosine, which can then be converted to various ceramides by five different ceramide synthases. The salvage pathway starts from degradation of sphingomyelin (SM) or glycosylated ceramides to ceramide, and degradation of ceramide to sphingosine (Sph), which is then secreted to the cytosol. Cytosolic Sph can be used to synthesize ceramide or S1P^3–5^. Among sphingolipids, S1P is well studied as it mediates diverse cellular processes, including cell growth, suppression of apoptosis, differentiation, angiogenesis and inflammation, and also serves in an autocrine and paracrine signaling via five different S1P receptors^6–8^. Additionally, S1P in the nucleus produced by sphingosine kinase 2 (sphk2) is known to control gene transcription^9^. S1P appears to play a role in the inflammation of typical steatohepatitis^10,11^, although the mechanism of its effects remain unknown.

KDSR is a key enzyme in the synthesis of sphingolipid. However, since KDSR was identified 20 years ago in yeast^12^, *in vivo* function of KDSR has been under-studied due to the lack of an animal model. We found progression of liver disease phenotype in the *kdsr* mutant zebrafish and we investigated the mechanism of disease pathogenesis in this paper. Given the well-conserved sphingolipid synthetic pathway in zebrafish and high protein homology with human KDSR, we expect that people who carry *KDSR* mutations may have liver disease. While recent human studies showed that mutations in *KDSR* are associated with keratinization disorder^13,14^, liver abnormalities in those patients have not been studied to date.

Zebrafish are a powerful model to study liver disease since their liver possess cells that are functionally analogous to those of mammals^15^ and have similar lipid metabolism to humans^16^. We previously discovered the novel zebrafish *kdsr*^*I105R*^ mutant that encodes a missense mutation in *3-ketodihydro-sphingosine reductase* (*kdsr*) from a forward genetic screening to identify mutants with post-developmental liver disease^17^. Here, we use the *kdsr*^*I105R*^ mutant to explore its role in the pathogenesis of hepatic injury. We found that accumulation of ceramides, Sph, and S1P resulted from activation of the lysosomal sphingolipid salvage pathway in the *kdsr*^*I105R*^ mutant. Additionally, we found that oxidative stress by elevation of mitochondrial β-oxidation and ER stress in the *kdsr*^*I105R*^ mutant can mediate mitochondrial cristae and liver injury. Through genetic interaction of *kdsr* and *sphk2* mutations, we also found that sphk2-mediated S1P accumulation is a key factor in both oxidative and ER stress in the *kdsr*^*I105R*^ mutant.

## Results

### *kdsr*^*I105R*^ mutant zebrafish developed progressive liver injury and hepatic injury during post-developmental stage

**Figure 1:**
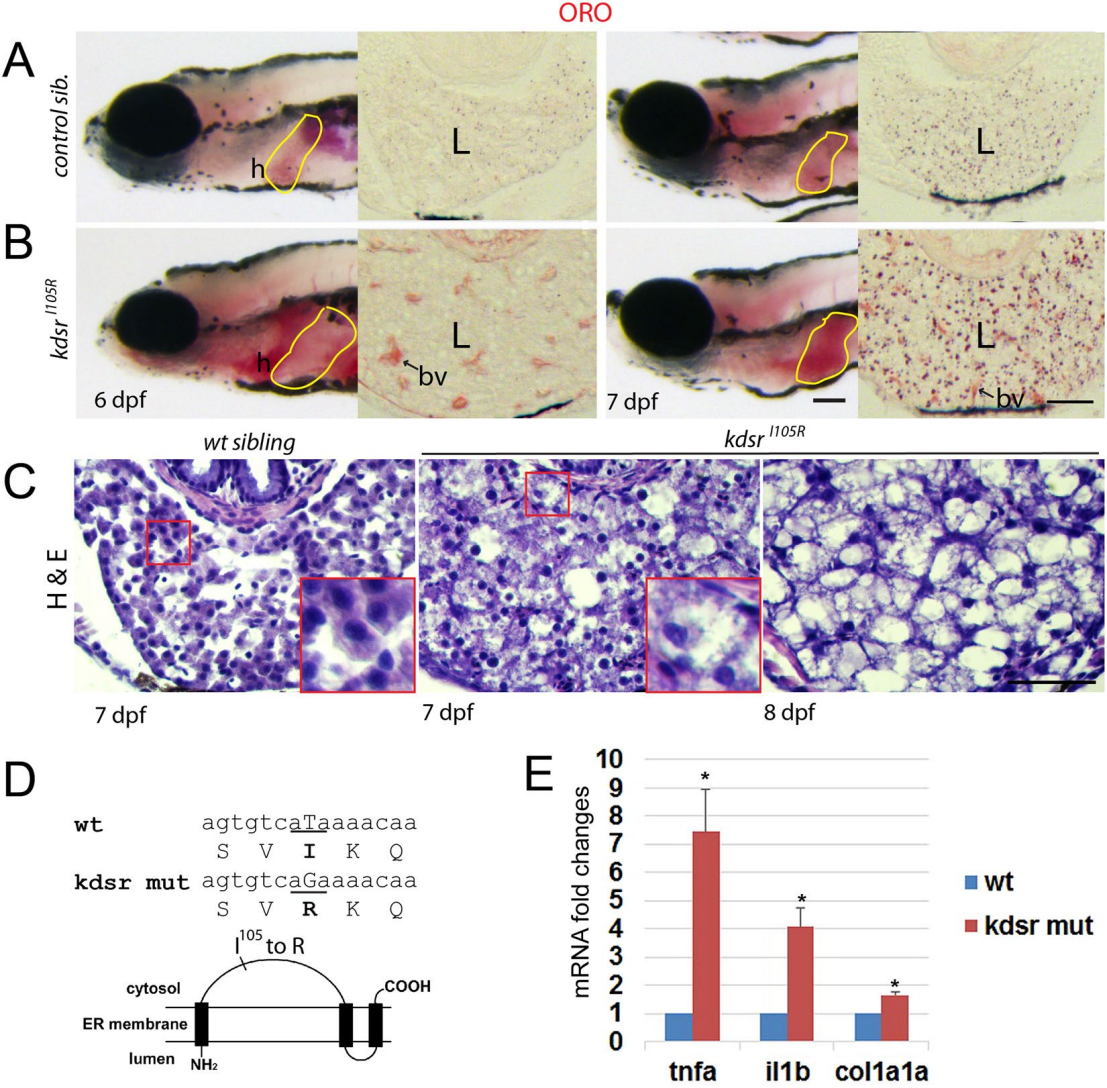
Progression of liver injury in the *Kdsr*^*I105R*^ zebrafish mutant. Whole mount oil-red O (ORO) staining in wild type control sibling (**A**, left) and *kdsr* mutant (**B**, left) at 6 days post fertilization (dpf) and 7 dpf. ORO staining performed in transverse section of the liver (L, right panels of A and B). N = 10 per each. Lipid accumulation in the heart (h) and blood vessel (bv) are marked in images of panel B. H & E staining results in control at 7 dpf (left), mutant at 7 dpf (middle), and mutant at 8 dpf (right) (C, n = 6 per each). The magnified area depicts hepatocyte ballooning in mutants at 7 dpf. Images shown are representative of at least 10 other zebrafish or livers, respectively. Scale bars = 100 µm (whole mount ORO staining in **A** and **B**), 40 µm (ORO staining on the liver section in **A** and **B**), and 100 µm (H&E staining in **C**). The predicted protein structure of kdsr and the locus of the mutated amino acid is shown in (**D**). Relative mRNA expression of tnfa, il1b, and col1a1a in the wild type and mutant siblings is shown in (**E**). *P ≤ 0.05.

From the previous forward genetic screening to identify zebrafish mutants with post-developmental liver defects^17^, we identified a mutant showing progression of liver defects ranging from hepatomegaly at 6 days post fertilization (dpf) to steatosis at 7 dpf, and to a more advanced hepatic injury thereafter (Fig. 1A–C). We identified causative mutation by using whole genome sequencing of normal looking siblings and homozygous mutants (Supporting Fig. 1). The mutant carried a missense mutation in *3-ketodihydrosphingosine reductase* (*kdsr*). The protein homology comparison with human KDSR showed that the zebrafish kdsr had high homology with human KDSR (81% identity and 93% positivity, Supporting Fig. 2). The secondary structure of human KDSR was previously reported^18^ and the missense mutation tyrosine to guanine (T to G) caused the isoleucine (I) to arginine (R) change at the 105 amino acid residue of kdsr (Fig. 1D). Hepatocyte ballooning occurred at 7 dpf and advanced hepatic injury was identified at 8 dpf (Fig. 1C). We also noticed that all homozygous mutant died between 8 to 10 dpf. The relative mRNA expression of genes associated with inflammation, including *tumor necrosis factor alpha* (*tnfa*) and *interleukin 1 beta* (*il1b*); and fibrosis, *collagen type 1 alpha 1a* (*col1a1a*) were significantly elevated in the *kdsr*^*I105R*^ mutants compared to controls (Fig. 1E). Thus, the *kdsr* mutant recapitulated characteristics found in hepatic injury in humans.

### Inhibition of fatty acid synthase activity exacerbated steatosis phenotype rather than suppressing steatosis in the *kdsr*^*I105R*^ mutant liver

**Figure 2:**
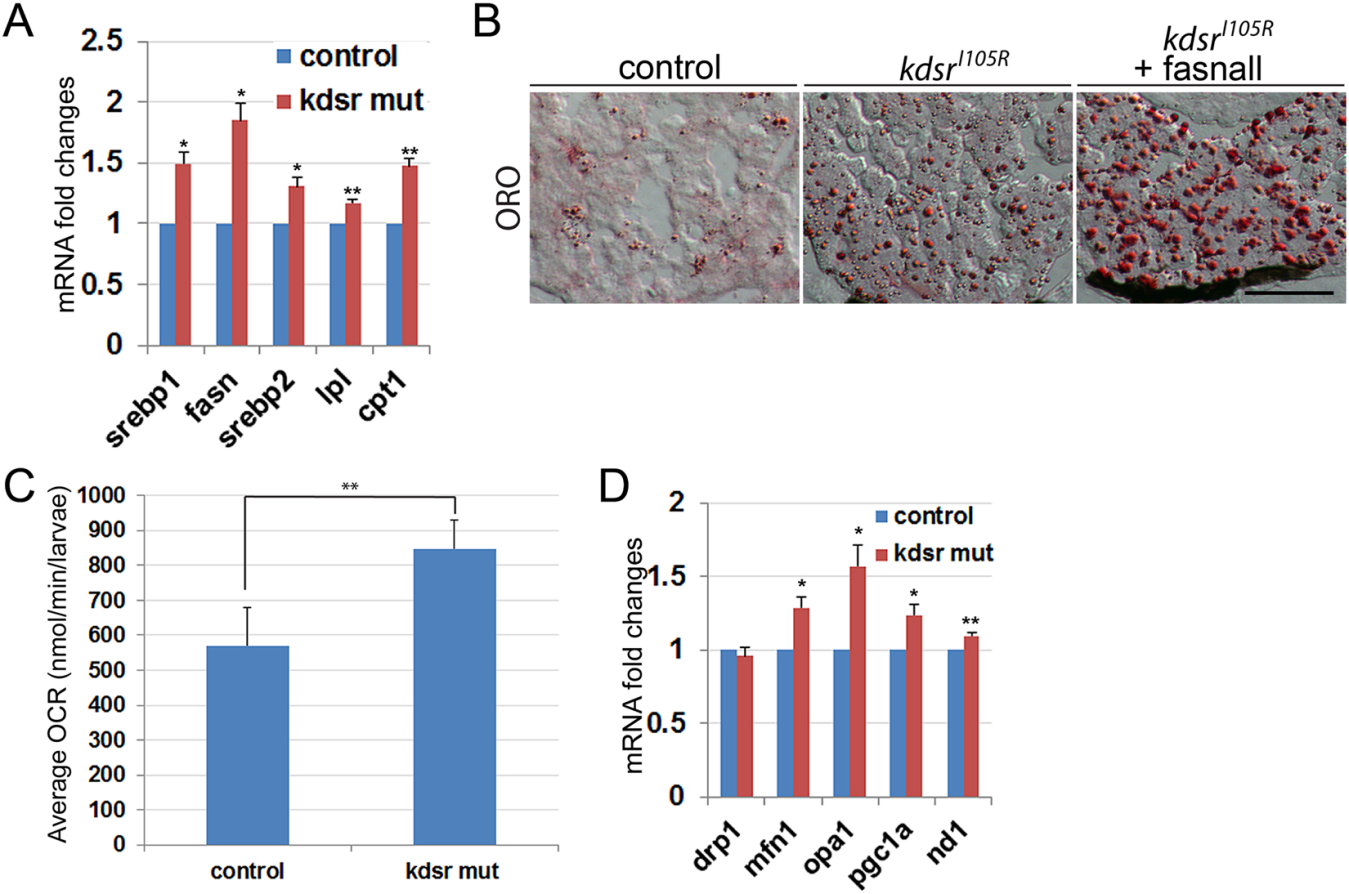
Lipid metabolism- and mitochondrial homeostasis-associated gene expression, and oxygen consumption analysis in larvae. Relative mRNA expression of genes associated with lipid metabolism include sterol regulatory element-binding protein 1 (*srebp1*), *fatty acid synthase* (*fasn*), *srebp2*, *lipoprotein lipase* (*lpl*), and *carnitine-palmitoyltransferase I* (*cpt1*) (**A**). Oil red O staining in control, *kdsr*^*I105R*^ mutant, and *kdsr*^*I105R*^ mutant siblings after fasnall treatment at 7 dpf; 1 µM fasnall treatment was performed from 4 dpf to 7 dpf (**B**). Images shown are representative of at least 10 in total. Scale bar in B = 100um. Average oxygen consumption rate was measured from 7 groups (3 larvae per group) of control and mutant siblings for 30 minutes (**C**). Relative mRNA expression of genes associated with mitochondrial homeostasis include *dynamin related protein 1* (*drp1*), *mitofusin 1* (*mfn1*), *optic atrophy type 1* (*opa1*), *peroxisome proliferator-activated receptor gamma coactivator 1-alpha* (*pgc1a*), and *NADH-ubiquinone oxidoreductase chain1* (*nd1*) (**D**). Error bars indicate standard deviation of the mean. *P ≤ 0.05, **P ≤ 0.005.

**Figure 3:**
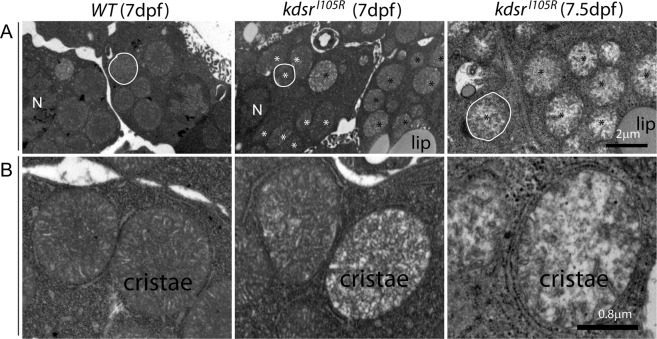
Transmission electron microscopy (TEM) imaging of hepatocytes. 8,000x magnification in wild type at 7 dpf (left), *kdsr*^*I105R*^ mutant at 7 dpf with mild phenotype (middle), and *kdsr*^*I105R*^ mutant at 7.5 dpf with severe defect (right) (**A**). One of representative mitochondria is outlined with white color. N, nucleus. Normal looking mitochondria and abnormal mitochondria were marked with white and black asterisks, respectively. 20,000x magnification images are shown in (**B**). Images shown are representative of 2 other animals. Scale bars = 2 µm (panel A), 0.8 µm (panel B). Lip, lipid drops.

To understand the mechanism of steatosis in the *kdsr*^*I105R*^ mutant, we analyzed mRNA expression of proteins involved in lipid metabolism (Fig. 2A). We found significant increases of *srebp1*, which regulates genes essential for lipogenesis, and *fasn*, a gene regulated by srebp1 that plays a role in palmitate synthesis from acetyl-CoA and malonyl-CoA. We also found significant increases of *srebp2*, which is essential for cholesterol biogenesis and the regulation of *lpl* expression, which encodes lipoprotein lipase, a key enzyme in lipid uptake (Fig. 3A). The hyperlipidemic phenotype in the *kdsr*^*I105R*^ mutant (Fig. 1B) may have activated the expression of *srebp2* and *lpl* expression to reduce plasma lipid level in the plasma rather than to cause liver steatosis. Since an increase in *fasn* expression may induce lipid accumulation in the liver, we treated mutants with fasnall, an inhibitor of fasn to determine whether the steatosis resulted from increase of lipogenesis. Interestingly, we found that the fasnall treatment exacerbated the steatosis phenotype in the *kdsr*^*I105R*^ mutant rather than suppressing lipid accumulation (Fig. 2B). This result suggests that *fasn* upregulation was required to facilitate mitochondrial β-oxidation, because palmitate is required for lipid transport to the mitochondria through cpt1, and one of the substrates of fasn, malonyl-CoA, is known to inhibit mitochondrial β-oxidation^19^. Elevation of *cpt1* expression further supports the enhancement of mitochondrial β-oxidation in the *kdsr*^*I105R*^ mutant (Fig. 2A), which is supported by the increase in oxygen consumption in the *kdsr*^*I105R*^ mutants compared to control siblings at 7 dpf (Fig. 2C). Analysis of genes expressed in association with mitochondrial homeostasis revealed a significant increase in *mfn1*, important for mitochondrial fusion, *opa1*, which plays a role in mitochondrial fusion and cristae stability, *pgc1a*, a master regulator of mitochondrial biogenesis^20^, and *nd1*, a gene expressed in the mitochondria and encodes a subunit of NADH dehydrogenase, while expression of *drp1*, a gene required for mitochondrial fission was not changed (Fig. 2D).

### Ultrastructural analysis of mitochondrial cristae in the *kdsr*^*I105R*^ mutant

Elevation of mitochondrial β-oxidation and oxygen consumption suggested that there may be accumulation of oxidative stress molecules in the mitochondria, which can induce mitochondrial injury. To test this possibility, we performed ultrastructural analysis wild-type control at 7 dpf and *kdsr*^*I105R*^ mutant livers at 7 dpf and 7.5 dpf. We found that there was accumulation of mitochondria with less dense cristae structures in hepatocytes of mutant at 7 dpf (Fig. 3A middle panel). There was also a more severely damaged and swollen mitochondrial phenotype in the *kdsr*^*I105R*^ mutant at 7.5 dpf (Fig. 3A right panel), suggesting progression of mitochondrial injury from less dense cristae damage to swollen and disrupted cristae phenotype. This could be a key factor for progression of liver phenotype from steatosis to hepatic injury phenotype in the *kdsr*^*I105R*^ mutant. Higher magnification of mitochondria (Fig. 3B), clearly showed cristae defects in the mutants. Activation of *opa1* (Fig. 2D) further supported damage of the cristae, because opa1 plays a role in stabilizing injured mitochondrial cristae^21^.

### *Kdsr*^*I105R*^ mutants accumulated sphingolipids through activation of the sphingolipid salvage pathway

**Figure 4:**
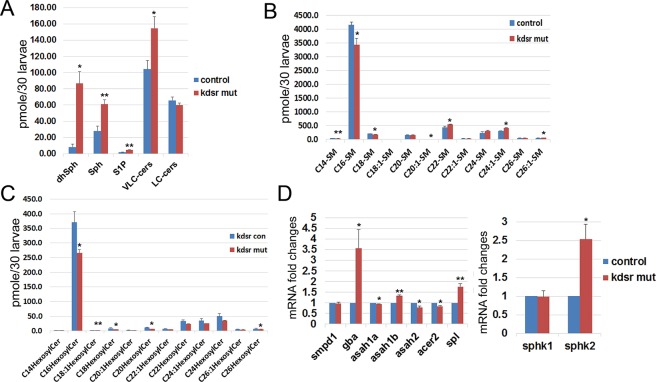
Activation of the sphingolipid salvage pathway in the *kdsr*^*I105R*^ mutant zebrafish. Accumulation of ceramides (cers), sphingosine (Sph) and sphingosine 1-phosphate (S1P) (**A**), decrease of SM (**B**) and hexosyl-ceramides (**C**) in the *kdsr* mutant larvae at 7 dpf. Thirty larvae were used per each assay and assays were performed in triplicate. Relative mRNA expression in sphingolipid salvage pathway components; *acid sphingomyelinase/sphingomyelin phosphodiesterase 1* (*smpd1*), *β-glucocerebrosidase* (*gba*), *acid ceramidase 1b* (*asah1b*), *neutral ceramidase 2* (*asah2*), *alkaline ceramidase 2* (*acer2*), and *sphingosine 1-phosphate lyase* (*spl*) (**D**, left). sphingosine kinase expression in the *kdsr*^*I105R*^ mutant; *sphingosine kinase 1* (*sphk1*)*, sphingosine kinase 2* (*sphk2*) (**D**, right). Error bars indicate standard deviation of the mean. *P ≤ 0.05, **P ≤ 0.005.

Because kdsr is a key enzyme for the *de novo* synthesis of sphingolipids, we expected the missense mutation of *kdsr* to affect sphingolipid synthesis. Sphingolipidomics analysis data indicated accumulation of downstream components of sphingolipids, suggesting that the missense mutation might cause gain of kdsr function (Fig. 4A). To test our hypothesis, we generated a *kdsr* null mutant by CRISPR/Cas9 gene targeting in zebrafish, carrying premature stop codon by deletion/insertion in target region at exon 3 (Supporting Fig. 3). The *kdsr*^*cri*^ null mutant showed the same phenotype as the *kdsr*^*I105R*^ mutant (Supporting Fig. 3A) and histological analysis of the *kdsr*^*cri*^ mutant showed that similar liver abnormality and steatosis observed in the *kdsr*^*I105R*^ mutant (Supporting Fig. 3B). Additionally, the sphingolipid profile mirrored that of the *kdsr*^*I105R*^ mutant (Supporting Fig. 3C,D). Complementation test was performed by crossing the heterozygous *kdsr*^*I105R*^ mutant and the *kdsr*^*cri*^ null mutant. We confirmed that the biallelic mutant (*kdsr*^*I105R/cri*^) also developed the same phenotype as the *kdsr*^*I105R*^ mutant (Supporting Fig. 4). We concluded that the missense mutation definitely induced loss of kdsr function. To understand how loss of kdsr function led to accumulation of downstream sphingolipids, we investigated the sphingolipid salvage pathway, which involves the degradation of SM and hexosyl- ceramides. SM species analysis showed significant decrease in the concentration of long chain SM species in the *kdsr*^*I105R*^ mutant, including C16-SM, the most abundant SM (>4000 pmole per sample), C14-SM, C18-SM and C20:1-SM in the *kdsr*^*I105R*^ mutant, while there were statistically significant increase of very long chain SM species such as C22-SM, C24:1-SM and C26:1-SM (Fig. 4B). We also found significant decrease of hexosyl-ceramides (glucosyl- or galactosyl-ceramides), including C16-, C18:1-, C18-, C20-, C26-Hexosyl-ceramides (Fig. 4C). This result suggests that C16-SM and hexosyl-ceramides were mainly used for the sphingolipid salvage pathway in the *kdsr* mutant zebrafish to compensate loss of sphingolipid de novo synthetic pathway. mRNA expression analysis showed a significant increase *gba*, which degrades glucosyl-ceramide into ceramides, *asah1b*, which is associated with lysosomal degradation of ceramides, and a decrease of *asah2*, a key enzyme in the plasma membrane, and *acer2*, which is found in the Golgi (Fig. 4D). Because SM is initially converted to ceramide by lysosomal acid sphingomyelinase encoded by *smpd1*, we also examined *smpd1* expression and found no significant change in the *kdsr*^*I105R*^ mutant (Fig. 4D). This result suggests that the lysosomal sphingolipid salvage pathway caused SM and hexosyl-ceramides degradation by transcriptional activation of *gba* and *asah1b* in the *kdsr*^*I105R*^ mutant, and resulted in the accumulation of ceramides, Sph and S1P in the mutant. We also found transcriptional activation of *sphk2* in the *kdsr*^*I105R*^ mutant, while no change was detected in *sphk1* expression (Fig. 4D). This result suggests that sphk2 is the major kinase involved in S1P accumulation in the mutant. In addition, mRNA expression of *spl*, the enzyme that mediates the degradation of S1P increased and possibly was induced to reduce accumulated of S1P in the *kdsr*^*I105R*^ mutant (Fig. 4A). Further, we analyzed gene expression of salvage pathway related in the *kdsr*^*cri*^ mutant to determine whether activation of sphingolipid salvage pathway is conserved in the *kdsr* null mutant (Supporting Fig. 3E). We found significant increases of *asah1b* and *spl* in the *kdsr*^*cri*^ mutant, which is same as in the *kdsr*^*I105R*^ mutant. Importantly, we found activation of *smpd1*, *gba*, *ash1a* and *acer2* in the *kdsr*^*cri*^ mutant, which suggesting *kdsr*^*cri*^ mutant may have higher activity in salvage pathway compared to *kdsr*^*I105R*^ mutant. Although both mutants have similar pathological defects, *kdsr*^*I105R*^ mutant may have minimal activity of *kdsr* function.

### Depletion of Sphk2 suppressed liver injury in the *kdsr*^*I105R*^ mutant

**Figure 5:**
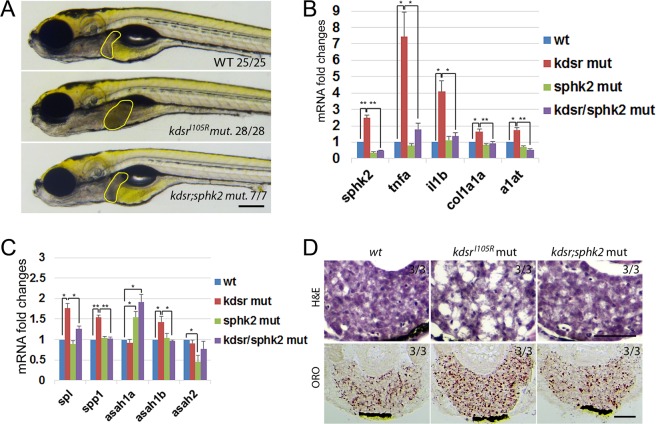
*Sphk2* deletion suppresses liver defects in the *kdsr*^*I105R*^ mutant. Images of live wild type (wt), *kdsr*^*I105R*^, and *kdsr*^*I105R*^*; sphk2*^*wc1*^ double mutant at 7 dpf (**A**). Scale bar = 0.25 mm. Relative mRNA expression of *sphk2* and other genes associated with inflammation (*tnfa*, *il1b*), tissue injury (*alpha1-anti-trypsin* (*a1at*)) and fibrosis (*col1a1a*) (**B**), sphingolipid salvage pathway related genes (**C**) in *wt*, *kdsr*^*I105R*^, *sphk2*^*wc1*^, and *kdsr*^*I105R*^; *sphk2*^*wc1*^ mutant siblings. *spp1*, (*S1P-phosphohydrolase 1*). Images of H&E and ORO staining in livers of *wt*, *kdsr*^*I105R*^ and *kdsr*^*I105R*^; *sphk2*^*wc1*^ double mutants (**D**, n = 6). Error bars indicate standard deviation of the mean. *P ≤ 0.05, **P ≤ 0.005. Scale bare in D = 100 µm.

A previous report showed that *sphk2* is the main sphingosine kinase in zebrafish embryo^22^. Because of transcriptional up-regulation of *sphk2* in *kdsr*^*I105R*^ mutant, and since accumulation of S1P has been reported to be involved in inflammation and steatohepatitis^10^, we tested whether the loss of sphk2 in the *kdsr*^*I105R*^ mutant can suppress liver defects. We used an *sphk2*^*wc1*^ mutant, a null mutant^23^. By crossing chimeric heterozygous (*kdsr*^*I105R/*+^*; sphk2*^*wc1/*+^) mutants, we were able to obtain double homozygous mutants, which exhibited complete suppression of liver defects seen in the *kdsr*^*I105R*^ mutant (Fig. 5A). Heterozygous *kdsr*^*I105R/*+^, *sphk2*^*wc1/*+^ mutants or homozygous *sphk2*^*wc1*^ mutant had normal livers (data not shown). The qPCR analysis demonstrated that genes associated with inflammation (*tnfa*, *il1b*), tissue injury (*a1at*) and fibrosis (*col1a1a*) were all significantly reduced in the double mutant (Fig. 5B). We also investigated effects of *sphk2* mutation on the sphingolipid salvage pathway. Significant suppression of mRNAs of *spl* and *spp1*, which functions to convert S1P to Sph, and *asah1b* was detected in *kdsr*^*I105R*^*; sphk2*^*wc1*^ double mutant, whereas no significant change was detected in *asah2* levels. The *sphk2*^*wc1*^ mutant showed significant decrease of *asah2* expression. However, *asah1a* gene expression was elevated in both *sphk2* mutant and *kdsr*^*I105R*^*; sphk2*^*wc1*^ double mutant (Fig. 5C). The qPCR results suggested that the salvage pathway is still activated in the *kdsr*^*I105R*^; *sphk2*^*wc1*^ mutant by elevating *asah1a* expression, a homolog of *asah1b* in zebrafish to maintain essential sphingolipids homeostasis for survival as additional compensatory mechanism. In addition, sphk2 depletion attenuated expression of genes (*spl*, *spp1*) involved in regulation of S1P levels. Thus, our results suggest that sphk2-mediated S1P accumulation might have a key role in the development of liver defects in the *kdsr*^*I105R*^ mutant. Histological analysis of *wt*, *kdsr*^*I105R*^ mutant and *kdsr; sphk2* double homozygous mutant showed that suppression of liver defects by sphk2 depletion in the *kdsr*^*I105R*^ mutant (Fig. 5D).

### Sphk2 played a key role in oxidative stress and ER stress in the *kdsr*^*I105R*^ mutant

**Figure 6:**
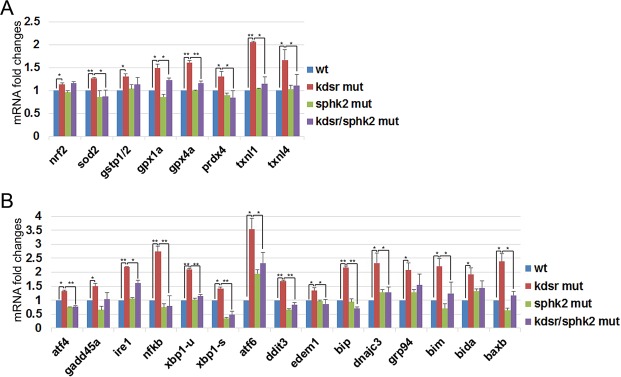
mRNA expression of genes associated with oxidative stress and ER stress. Relative mRNA expression of oxidative stress-related genes; *nuclear factor erythroid 2 - related factor 2* (*nrf2*), *superoxide dismutase 2* (*sod2*), *glutathione S transferase pi 1* and *2* (*gstp1/2*), *glutathione peroxidase 1a/4a* (*gpx1a, gpx4a*), *peroxiredoxin 4* (*prdx4*), and *thioredoxin-like 1/4* (*txnl1, txnl4*) (**A**) and ER stress/UPR response related genes; *activating transcription factor 4* (*atf4*), *growth arrest and DNA-damage-inducible 45 alpha* (*gadd45a*), *inositol-requiring enzyme 1* (*ire1*), *nuclear factor kappa-light-chain-enhancer of activated B cells* (*nfkb*), *X-box binding protein 1* (*xbp1*), spliced form of *xbp1* (*xbp1-s*), *activating transcription factor 6* (*atf6*), *DNA damage-inducible transcript 3* (*ddit3*), *ER degradation-enhancing alpha-mannosidase-like 1* (*edem1*), *binding immunoglobulin protein* (*bip*), *DnaJ homolog subfamily C member 3* (*dnajc3*), *glucose-regulated protein 94* (*grp94*), *Bcl2 interacting mediator of cell death* (*bim*), *BH3-interacting domain death agonist a* (*bida*), and *Bcl2 associated x b* (*baxb*) (**B**). Error bars indicate standard deviation of the mean. *P ≤ 0.05, **P ≤ 0.005.

Both oxidative stress and ER stress are major contributors to mitochondrial injury and liver disease^24,25^. We first analyzed the expression of genes activated by oxidative stress. We found significant elevation of oxidative stress-related genes in the *kdsr*^*I105R*^ mutants compared to controls, suggesting that liver injury may be related to increased oxidative stress (Fig. 6A). Importantly, significant elevation of oxidative stress-related genes in the *kdsr*^*I105R*^ mutant were suppressed by introducing the *sphk2* null mutation into the *kdsr*^*I105R*^ mutant (Fig. 6A). To investigate whether ER stress also contributed to liver defects in the *kdsr*^*I105R*^ mutant, we analyzed the expression of genes associated with ER stress response in each control and mutant sibling, including *atf4*, and its target gene *gadd45a*. The *Ire1*, one of upstream component of *nfkb* transcription that functions as endonuclease to process *xbp1*, resulting in increased spliced form of *xbp1* (*xbp1-s*) under ER stress condition. The *atf6*, and targets including *xbp1*, *ddit3*, *edem1*, *bip*, *dnajc3*, and *grp94* were also analyzed. Additionally, *bim*, *bida*, and *baxb* were analyzed, which were known to be associated with ER stress-induced apoptosis through mitochondrial injury. Especially, *bip*, *dnajc3*, and *grp94*, molecular chaperons activated by unfolded protein folding response, are a main cause of ER stress. All of the tested genes in the ER stress pathway were all elevated in the *kdsr*^*I105R*^ mutant (Fig. 6B). Although transcriptional activation of *ire1* in the *kdsr*^*I105R*^ mutant was not suppressed by *sphk2* mutation, significant decrease of *xbp1-s* was found in both the *sphk2*^*wc1*^ mutant and *kdsr*^*I105R*^*; sphk2*^*wc1*^ double mutant. This result suggests that *sphk2* expression was required for endonuclease activity of *Ire1*. Transcriptional activation of *atf6* was found in the *sphk2*^*wc1*^ mutant; however, sphk2 depletion limited the activation of *atf6* in the *kdsr*^*I105R*^ mutant. Our result showed that even partial suppression of *atf6* by *sphk2* knockout was sufficient to suppress downstream target gene transcriptions including *ddit3*, *edem1*, *bip*, *dnajc3*, and *grp94*. These data suggest that sphk2 is a key mediator in the elevation of oxidative stress and ER stress in the *kdsr*^*I105R*^ mutant.

### Depletion of glutathione in the *kdsr*^*I105R*^ mutant

**Figure 7:**
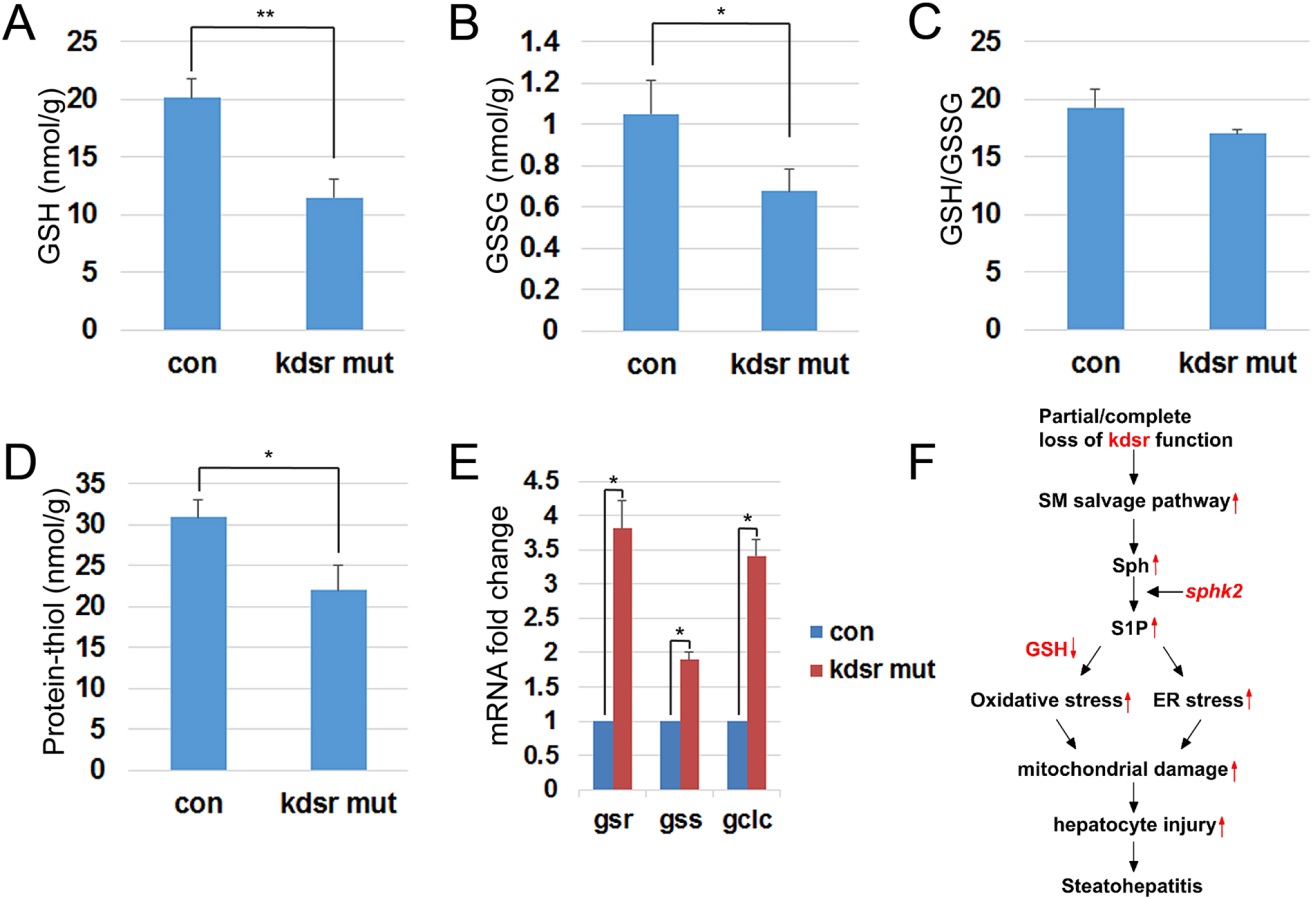
GSH, GSSG, and protein-thiol levels in control and *kdsr*^*I105R*^ mutant. Quantification of GSH (**A**), GSSG (**B**), GSH to GSSG ratio (**C**), and protein-thiol (**D**) in control and *kdsr*^*I105R*^ mutant. Three groups of control and mutant were used for analysis and each group contains 5 larvae. Relative mRNA expression of *glutathione reductase* (*gsr*) and *glutathione synthetase* (*gss*) in control and mutant (**E**). The working model illustrating that deleterious mutations in *kdsr*^*I105R*^ induced transcriptional upregulation of *sphk2*, resulted in S1P accumulation. S1P accumulation elevated oxidative stress and ER stress mediated mitochondrial injury, hepatic injury and then steatohepatitis. GSH depletion might sensitize *kdsr*^*I105R*^ mutant to oxidative stress (**F**). Error bars indicate standard deviation of the mean. *P ≤ 0.05, **P ≤ 0.005.

Glutathione (GSH) is a key antioxidant that protects cellular components from oxidative stress and injury. A reduction in the ratio of GSH to GSSG indicates that cells have increased susceptibility to oxidative stress^26,27^. The increase in mRNA expression of components of the redox pathway (Fig. 6A) in the *kdsr*^*I105R*^ mutant raised the possibility of severe oxidative stress. Therefore, we measured GSH, GSSG, and protein-thiol levels in the *kdsr*^*I105R*^ mutant. We found a significant reduction in both GSH and GSSG levels (Fig. 7A,B). Although the GSH/GSSG ratio was not significantly decreased, the decrease in total GSH levels suggests that the *kdsr*^*I105R*^ mutant might have limited capacity to protect proteins from ROS induced damage (Fig. 7C). Further, a significant decrease in the protein-thiol levels suggested accumulation of oxidized proteins in the *kdsr*^*I105R*^ mutant (Fig. 7D). In aggregate, these data suggest that GSH depletion/oxidative stress may play critical role in hepatocellular injury found in the *kdsr*^*I105R*^ mutant.

To understand the mechanism of GSH depletion, we examined mRNA expression of gsr, an enzyme that converts GSSG to reduced GSH. We also examined genes associated with GSH synthesis including gss, which synthesizes GSH from r-glutamylcysteine and glycine; and gclc, which synthesizes r-glutamylcystein (Fig. 7E). Collectively, the genes involved in GSH synthesis were elevated in the *kdsr*^*I105R*^ mutant, suggesting that GSH depletion was not a result of downregulation of those enzymes, and might be caused by limitation of substrate such as cysteine. Further, we treated control and mutant larvae with n-acetyl cysteine (NAC) to address whether NAC treatment can induce GSH synthesis and reduce ROS in *kdsr* mutants. The result showed that NAC treatment did not increased GSH level in both control and mutant larvae, which suggests that NAC treatment might not be effective in elevating GSH levels *in vivo* in zebrafish. However, we found a significant decrease of the oxidized form of GSH (GSSG) in control siblings compared to mutants. As a result, NAC treatment elevated GSH/GSSG ratio in the control larvae, which led to ROS a decrease in ROS. However, NAC treatment was not effective in elevating GSH/GSSG ratio and suppressed ROS levels in the *kdsr* mutants (Supporting Fig. 5).

### Hepatocellular injury in heterozygous *kdsr*^*I105R/*+^ mutant adults

**Figure 8:**
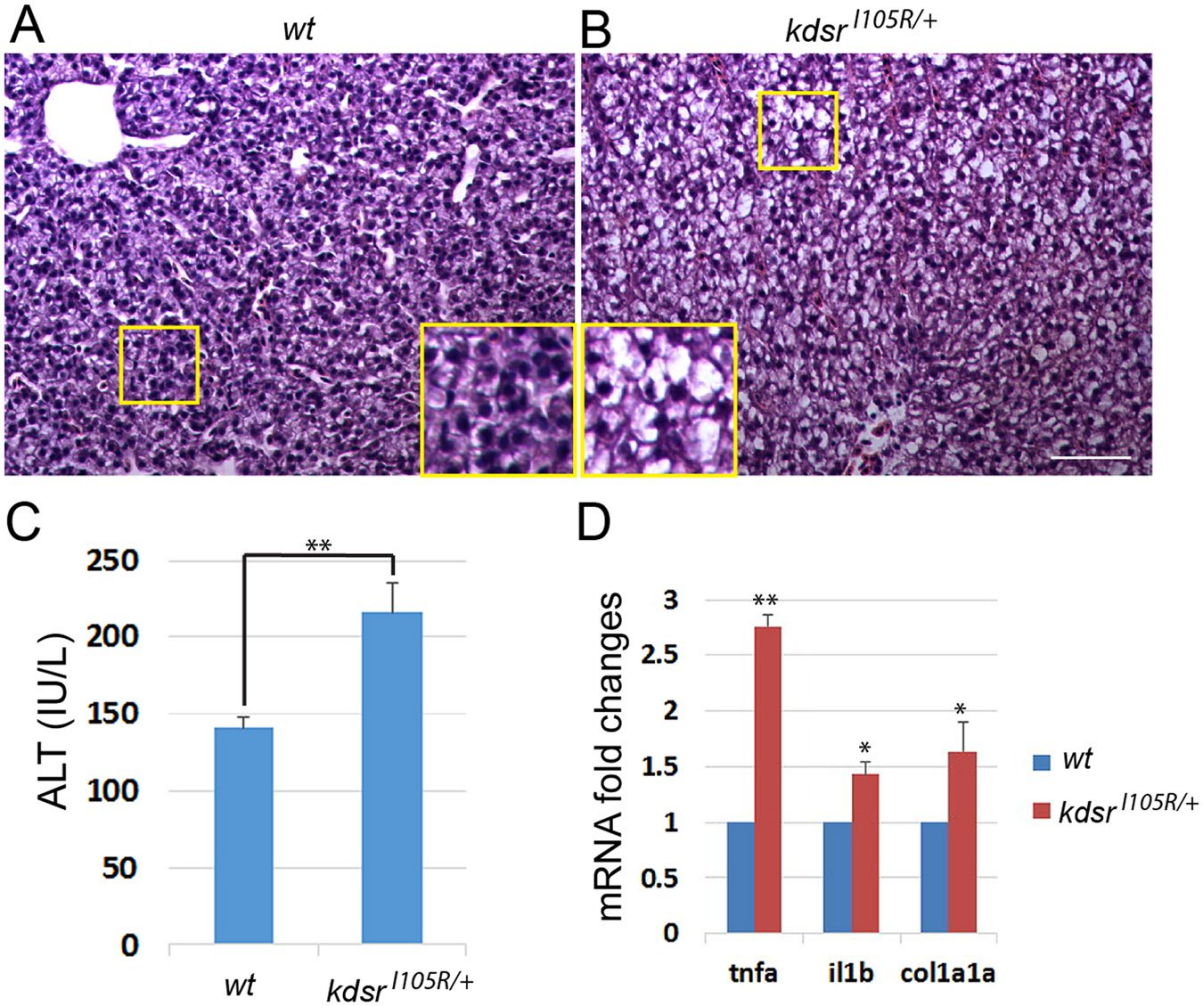
Predisposed liver injury in the *kdsr*^*I105R/*+^ adult zebrafish. H & E staining of wild type liver (**A**) and heterozygous *kdsr* mutant liver (**B**). The inset depicts high resolution of the identified areas. Images shown are representative of at least 10 in total. Scale bar for A and B is 100 µm. Serum ALT test (**C**) in wild type and heterozygous kdsr mutant (n = 3). Relative mRNA expression of *tnfa*, *il1b*, and *col1a1a* (**D**) and *sphk2*, *asah1a*, *asah1b*, and *asah2* (**E**) in wild type and *kdsr*^*I105R/*+^ mutants (n = 3). Error bars indicate standard deviation of the mean. *P ≤ 0.05, **P ≤ 0.005. ALT, alanine aminotransferase.

To investigate whether the heterozygous *kdsr* mutation alone leads to hepatocellular injury in adult zebrafish, we also analyzed livers of wild type and *kdsr*^*I105R/*+^ heterozygous mutant siblings. Ballooning of hepatocyte phenotype (Fig. 8A,B) and an increase of serum alanine-aminotransferase (ALT) levels (Fig. 8C) were identified in adults. Additionally, mRNA levels of *tnfa*, *il1b* and *col1a1a* expression were increased in the livers of *kdsr*^*I105R*^ heterozygous mutants (Fig. 8D), similar to homozygous mutant larvae (Fig. 1E). To determine whether the heterozygous mutation also can enhance the sphinggolipid salvage pathway similar to homozygous mutant larvae, we analyzed genes involved in the sphingolipid salvage pathway. qPCR analysis revealed significant elevation of *sphk2* and *asah1a* mRNAs in the liver (Fig. 8E). These data suggest that the liver injury phenotype of the *kdsr*^*I105R/*+^ adult may also result from S1P accumulation in a fashion similar to that of the *kdsr*^*I105R*^ homozygous mutant.

## Discussion

In this study, we have found that a novel *kdsr*^*I105R*^ mutant had progression of liver disease from hepatomegaly to hepatic injury. Since *KDSR* deficiency was discovered about one year ago, liver abnormalities have not been addressed in *KDSR* human patients. It is possible that mutations in patients were hypomorphic, and biallelic mutations might not be enough to activate the sphingolipid salvage pathway. Our working model (Fig. 7F) showed that suppression of kdsr function by either homozygous or heterozygous mutations can enhance the sphingolipid salvage pathway, and that excess Sph is mainly phosphorylated by sphk2. Increase of S1P levels in turn might trigger oxidative stress via elevation of mitochondrial β-oxidation. Additional ER stress could be attributed to mitochondrial damage. Both stresses associated with liver disease were suppressed by loss of sphk2 function. Significant decrease of GSH in the *kdsr* mutant might sensitize *kdsr* mutant larvae against to oxidative stress. Mitochondrial injury could be upstream event preceding hepatic injury in the homozygous *kdsr*^*I105R*^ mutant larvae and heterozygous *kdsr*^I105R/+^ mutant adults. Thus, we found how kdsr dysfunction can induce hepatic injury phenotype in both homozygous *kdsr* mutant larvae and heterozygous *kdsr* mutant adult fish. Our results suggest that genetic variant causing decrease of kdsr activity could be an underlying risk factor for development of liver disease and people who carry deleterious mutations in adult might be highly susceptible to liver disease such as steatohepatitis or advanced liver disease such as fibrosis or hepatocellular carcinoma.

The progressive liver injury phenotype identified here was associated with progression of mitochondrial injury phenotype from less dense cristae structure to swollen mitochondria. The progressive liver defect was similar to the defect observed in the *electron transfer flavoprotein alpha* (*etfa*) mutant, a zebrafish model of multiple acyl-coA dehydrogenase deficiency^28^, suggesting that mitochondrial defects could be key factor in liver phenotype in the *kdsr*^*I105R*^ mutant.

The homoeostasis of cellular sphingolipids are tightly controlled by both a *de novo* synthesis (initiated from palmitoyl-CoA and serine to produce ceramides) as well as a salvage catabolic pathway (initiated from complex sphingolipids such as glycosylated ceramides or sphingomyelins to produce ceramides) through the regulation of the intra-cellular levels of ceramide. Ceramide is the central molecule in the sphingolipid metabolic pathway that can be converted to various sphingolipid species^29^. Diet-induced alterations in ceramide via both *de novo* and salvage sphingolipid synthesis demonstrate that nutrition has the ability to alter sphingolipid metabolism and in turn downstream signaling pathways^30^. Enzymes of the salvage pathway have been implicated in dietary manipulations of ceramide levels. Ceramide acts as the central molecule in the sphingolipid metabolic pathway. A previous study showed that radio-labeled palmitate was found in the dihydrosphingosine, and then in the ceramides via *de novo* synthesis. However, they also found ceramides produced via salvage pathway at the same time. Thus, palmitic acid treatment can enhance ceramide formation through the both *de novo* and salvage pathway^31^. Furthermore, administration of high fat diet enhanced mRNA expression and activity of acid sphingomyelinase and neutral sphingomyelinase in rat liver^32^ and mouse adipose tissue^33^. Pharmacological inhibition of acid sphingomyelinase inhibited ceramide induction by high fat diet in plasma and adipose tissue in mice^34^. However, compared to studies examining *de novo* sphingolipid synthesis, regulation of the salvage pathway has not been well-studied. Future experimentation that include modeling of sphingolipid metabolism can help understand the role the salvage pathway in mammals. To determine impact of external feeding in sphingolipid salvage pathway in zebrafish after consumed own egg yolk, we performed a diet experiment in control and *kdsr* mutant siblings at 7dpf. We found egg yolk feeding induced significant increase of gene expression involved in salvage pathway in both control and mutants (Supporting Fig. 6). Additionally, this result suggested that activation of salvage pathway in the *kdsr* mutant was not induced by starvation.

Loss of kdsr function should in theory inhibit *de novo* synthesis of sphingolipids and deplete downstream sphingolipids. Due to the current lack of LC-MS methods to quantify the substrate of kdsr, 3-keto-dhSph, it is expected that significant amount of 3-keto-dhSph would be accumulated in the mutant. As 3-keto-dhSph and Sph are isomers, the *kdsr* mutant may instead produce 3-keto-dihydroceramide, which may have inhibitory effect on dihydroceramide desaturase, the enzyme that generates ceramide from dihydroceramide. This hypothesis may explain how endogenous dhSph and dihydroceramide, possibly transported from egg yolk accumulated in the *kdsr* mutant (Supporting Fig. 7). As 3-keto-sphingolipids have not been sufficiently studied, there is unfortunately no quantification methods available yet. Further, future studies on sphingolipids with the 3-keto moiety will be necessary to answer this question. In this study, we found that inhibition of the kdsr function activated the sphingolipid salvage pathway possibly to compensate for the inhibition of the de novo synthesis pathway. As a result, Sph, S1P and ceramides may have accumulated in the *kdsr*^*I105R*^ mutant. Because the larvae could have used the egg yolk as nutrient by 6 dpf, probably there was no external source of sphingolipids without feeding the larvae, thus the salvage pathway would be the only way to produce downstream sphingolipid species. Previous studies have shown that accumulation of ceramides may induce steatohepatitis in^35–38^. However, our genetic study of the interaction of *sphk2* and *kdsr* mutation suggested that S1P is likely the cause of the observed liver phenotype (Fig. 5).

Sphk1 was found to be necessary for S1P-mediated steatohepatitis in a high fat diet-induced liver disease in mice; however, the role of SphK2 was not investigated^10^. Notably, our findings raised the possibility that sphk2 is the major sphingosine kinase involved in steatohepatitis (Fig. 5B) and this finding is consistent with a previous report that showed sphk2 functions as the main sphingosine kinase for S1P production in zebrafish^22^. Importantly, a significant increase of SPHK2 expression was found in both steatosis and steatohepatitis cases of multiple patients^39^. Thus, an important finding from the current study is that *sphk2* may play a key role in the development of hepatic injury associated with *kdsr* mutation and then steatohepatitis. The effect of high fat diet in the *kdsr*^*I105R*^ mutant remains to be determined.

Cellular GSH maintains the oxidation status of thiols in critical proteins and defends cells against to reactive oxygen species by its reducing capacity^40^. GSH depletion might reduce the buffering capacity of GSH against oxidative stress, which plays a key role in the aging process and the pathogenesis of many diseases^41,42^. Based on mRNA expression analysis, enzymes responsible for GSH synthesis and reduction are highly upregulated in the *kdsr* mutant, although total GSH level is significantly lower than control siblings (Fig. 7). This result suggests that GSH depletion may occur by depletion of a substrate(s) for GSH synthesis. Further investigation will be necessary to address the mechanism of GSH depletion. We propose that cysteine depletion might affect GSH levels in the *kdsr* mutant, since cysteine is tightly regulated in the liver for both GSH synthesis and protein synthesis^43^. We tested whether NAC treatment can elevate GSH synthesis, but NAC treatment did not elevate GSH in both control and *kdsr* mutants (Supporting Fig. 5). A previous study showed that NAC concentrations would have to exceed 1 mM, which is therapeutically unattainable *in vivo* to achieve maximum rates of GSH synthesis^44^. The NAC treatment might not be enough to elevate GSH synthesis in zebrafish same as in human. In this paper, we were not able to make a conclusion whether cysteine depletion is the main cause of GSH depletion, because sub-lethal dose of the NAC treatment did not elevate GSH levels *in vivo*. Further investigation will be necessary to determine the mechanism of GSH depletion in the *kdsr* mutant in the future.

Collectively, our findings indicate that kdsr deletion leads to compensatory activation of the sphingolipid salvage pathway and S1P accumulation, which can result in increased mitochondrial activity, oxidative stress, and ER stress and subsequent hepatocellular injury. The data point to the possibility that kdsr could be a novel genetic risk factor for steatosis and liver injury.

## Methods

### Animals

All methods of this article were performed in accordance with relevant guidelines and regulations of the NIH Guide for the Care and Use of Laboratory Animals and Medical University of South Carolina’s Division of laboratory animal resources (DLAR). All experiments on zebrafish were approved by the Institutional Animal Care and Use Committee (IACUC) of the Medical University of South Carolina (IACUC protocol #3364).

The zebrafish strain used in this study was AB/TU. Adults were maintained at 28.5 °C and fed twice a day with brine shrimp and Tetramin flake (Tetra US, Blacksburg, VA). Embryos were obtained from natural mating and raised at 28.5 °C in egg water (0.3 mg of sea salt/L). *Kdsr*^*I105R*^ and *sphk2*^*wc1*^^23^ lines were outcrossed with wild type AB/TU at least five times to reduce additional background mutations and maintenance. The *kdsr*^*I105R*^ mutant were genotyped using 5′-GTGGTTCTTTGCATTTCTGTTGATGT-3′ and 5′-ATGGCGAAAGGATTTTATGAATTGTTAAACATAC-3′, 30 cycles of PCR with 56 °C annealing temperature and then digested with Hpy188I (R0617, NEB, inc.) for 1 hr. *sphk2*^*wc1*^ was genotyped as described in the previous paper^23^. A *kdsr* null mutant was generated by CRISPR/Cas9 gene targeting in the laboratory. The guide RNA for *kdsr* was designed to target exon 3 of *kdsr* (5′-GGTTCAGGCTAAGAAAGAAGTGG-3′); T7-gRNA-kdsr nucleotides (5′-GAAATTAATACGACT CACTATAGGTTCAGGCTAAGAAAGAAGGTTTTAGAGCTAGAAATAGCAAGTTAAAAT) were used as template for gRNA synthesis. 50 to 100 pmole of guide RNA and 100 pmole of Cas9 RNA were co-injected at 1 cell-stage eggs. Injected embryos were raised and outcrossed with wild type to produce progeny. Each of the progenies was genotyped using 5′-GGTGTTACACAATTTGAAAACCATTTACC ACTG-3′ and 5′- TCCTTTGTTAAAGACATACATACT TGCTTATC-3′ primers, and deletions were confirmed by sequencing. Identified founder fish was used to generate stable lines. Siblings were used as controls.

### Whole genome sequencing

Genomic DNA from 10 normal siblings and 10 homozygous mutants were used as template DNAs for whole genome sequencing. The MUSC Sequencing Core performed sequencing of samples using an Illumina HiSeq2500 Platform with 150 bp paired-end reads, resulting in approximately 10 fold genomic coverage. The sequencing results were uploaded to the SNPtrack Mapping server (http://genetics.bwh.harvard.edu/ snptrack/), and mapped mutations re-confirmed by sequencing and genotyping of individual mutants. The sequencing results were submitted to NCBI Short Read Archive (SRA) database under the BioSample accessions SAMN10247386 and SAMN10247387.

### Oil Red O (ORO) Staining

For whole mount staining at the larval stage, we used the method previously described^28^. Briefly, larvae were fixed in 4% PFA overnight. The same numbers of control and mutant larvae (5 to 10 larvae each) were processed together in the same tube. After staining, larvae were briefly rinsed in PBS-Tween and fixed in 4% PFA for 10 minutes. Larvae were mounted in glycerol prior to imaging. For ORO staining on transversely sectioned larvae, frozen sections with 10 µm thickness were dried at room temperature for 5 minutes. 150 µl of working ORO solution was added to slides and stained for 30 seconds. They were then washed with distilled water and mounted using 75% glycerol.

### H&E staining

Embryos were fixed in 4% paraformaldehyde from overnight to two days at 4 °C. Fixed embryos were embedded in 1.2% agarose/5% sucrose, and saturated in 30% sucrose at 4 °C for 1 to 2 days. Blocks were frozen using liquid nitrogen. 10 µm sections were collected on microscope slides using a Leica cryostat. For adult liver histology, truncated bodies were fixed in 4% paraformaldehyde from overnight to two days at 4 °C and processed for embedding in paraffin. Paraffin sections were used for H&E staining, which was conducted in the Histo-Core lab at Medical University of South Carolina (MUSC). Images were taken with AxioCam ICC3 attached to Zeiss Axio-Imager M2.

### Blood preparation and alanine aminotransferase (ALT) measurements

Zebrafish blood was obtained by minimally invasive blood collection method using a heparinized needle. The site for blood collection is along the body axis and posterior to the anus in the region of the dorsal aorta. Blood was collected from adult zebrafish, 20 hours after feeding and diluted 1:10 in PBS. The average volume of blood collected from a three-individual fish (average body weight = 0.6 g) was 25 µL. Plasma was separated by centrifugation for 15 minutes at 2,000 × g using a refrigerated centrifuge. 10 µl of plasma were transferred to 96-well plate and ALT was measured using a microplate-based ALT activity assay kit (Pointe Scientific, Cat. A7526).

### Oxygen consumption assay of zebrafish larvae

Larval average oxygen consumption rate was determined using a sensor-dish reader (SDR) system (Loligo Systems, Viborg, DEN). Recordings were made once every 5 minutes for 1 hour using PreSens-SDR_v38 software (Loligo Systems, Viborg, DEN). Each well of 24-well optical fluorescence glass sensor microplate was filled with 125 µl of egg water and pre-ran for 20 minutes at 24 °C room. Three larvae were placed into wells of a 24-well plate, and then the plate was immediately sealed using parafilm and a silicone gel pad cover. Using PreSens–SDR_v38 software, dissolved oxygen amount was measured every 3 minutes for 30 minutes. Average oxygen consumption rate (nmol/L/min/fish) was calculated from change in O_2_ concentration over time. Data presented are from 7 measurements made with 3 larvae of each control and mutant siblings.

### Lipid analysis

Three sets of 7 day-old control siblings (n = 30) and mutant siblings (n = 30) were anesthetized and collected in 15 ml tube and kept in −80 °C before submission to LC/MS/MS analysis at the MUSC Lipidomics core facility as previously described^45,46^.

### Immunofluorescence Staining

To avoid variation of staining intensity, 3 of each control and mutant larvae were always processed together on the same glass slide. Slides were concurrently processed in Sequenza™ Slide Rack (Tedpella, Cat. 36105). Frozen sections were rehydrated in 1x PBS for 10 min at room temperature and blocked in 5% sheep serum in PBS for 2 hours. Sections were incubated with primary antibody to sod2 (Genetex, Cat. GTX124438, dilution 1:300) overnight at 4 °C, rinsed for 30 minutes (10 min × 3 times) with 1x PBS and then incubated for 2 hours with Alexa Fluor conjugated goat anti-rabbit secondary antibody. Sections were then washed with 1X PBS for 30 minutes and mounted in Vectashield with DAPI (Vector laboratories). Images were acquired using Zeiss Axiovert 200 M microscope with Zeiss AxioCam MRm and Hamamathu digital camera. Digital images were processed using Adobe Photoshop CS5 and Adobe illustrator CS5. All images received only minor modifications with control and mutant sections always processed in parallel.

### Transmission Electron Microscopy

Zebrafish were immersed in 2.5% glutaraldehyde in phosphate buffer, pH 7.4, overnight. Briefly, ultra-thin sections (70 nm) were positioned on copper grids, and stained with uranyl acetate and lead citrate for 10 minutes each. The sections were viewed on JEOL 1010 electron microscope at 80 kv in various magnifications. The images were captured with a Hamamatsu camera. Whole processes were performed in the Electron Microscopy core facility at the Medical University of South Carolina.

### Quantitative polymerase chain reaction (qPCR)

Oligo-dT primed complementary DNA were prepared from total RNA isolated with Trizol® Reagent (Invitrogen, Cat. 15596-026) using superscript III First-Strand kit (Invitrogen, Cat.18080-051). Real-Time qPCR was performed with Bio-rad, CFX96 Real-time system with 1 cycle of 98 °C for 30 seconds, 45 cycles of 95 °C for 15 seconds, and 60 °C for 30 seconds using 50 ng cDNA, 4 pmoles of each gene-specific primers per 20ul reaction (Supporting Table 1), and SsoAdvanced™ Universal SYBR® Green Supermix (Bio-rad, Cat. 172–5274). We used qPCR primers that were either used in previous studies in zebrafish^47–54^ or designed and tested in our lab (Supporting Table 1). *Glyceraldehyde-3-phosphate dehydrogenase* (*gapdh*) was used as reference, and relative quantification was calculated using double delta Ct method. The qPCR was run in at least triplicate for each gene. Total RNA were isolated from 20 control siblings and 20 *kdsr*^*I105R*^ homozygous mutant larvae. For multiple comparisons between *wild type*, *kdsr*^*I105R*^, *sphk2*^*wc1*^, and *kdsr*^*I105R*^*; sphk2*^*wc1*^ double homozygous mutants, *kdsr*^*I105R*^*; sphk2*^*wc1*^ double heterozygous mutants were crossed and siblings were anesthetized in 0.016% ethyl 3-aminobenzoate methanesulfonate salt (MS-222, Sigma-aldrich, E10521) with Ringer’s solution. Tips of tails were dissected for genotyping and bodies were kept in −80 °C. After genotyping of sibling larvae at 7 dpf, 5 of each wild type, single and double mutants were collected and total RNA were extracted for cDNA synthesis.

### Measurement of intracellular reduced thiol levels

Triplicated groups of control and mutant larvae, each contained 5 larvae at 7 dpf stage were lysed in 75 ul of ice-cold lysis buffer [50 mM Tris-HCl (pH 7.5), 150 mM NaCl, 1% Triton, 1 mM EDTA, 1 mM EGTA, plus a protease inhibitor cocktail (Roche/Sigma)]. 2ul of each lysate were immediately subjected to the reduced thiol measurement by using thiol fluorescent probe IV (Millipore) as previously described^55^. Fluorescent intensities were detected at 400Ex/465Em by Microplate Reader. The thiol fluorescent probe IV with lysis buffer was used as a negative control.

### Measurement of GSH and GSSG levels

Quantitative determinations of GSH and GSSG levels were performed using the enzymatic-recycling method^56^. Triplicated group of each control mutant larvae were subjected to lysis. Each group contains 5 larvae at 7 dpf. Protein in the extracts from 5 larvae was precipitated by sulfosalicylic acid and the supernatant was then divided into two. For reduced GSH, the supernatant was incubated with the thiol fluorescent probe IV, and fluorescent intensities were measured at 400Ex/465Em. For total GSH (GSH + GSSG), the supernatant was neutralized by triethanolamine and incubated with the reduction system (containing NADPH and glutathione reductase) at 37 °C for 20 min. GSSG was calculated based on the results from reduced GSH and total GSH; the ratio of $GSH/GSSG=\frac{[GSH]}{([TOTAL\,GSH]-[GSH])\,/\,2}$.

### Statistical tests

All evaluations were performed with MS Excel software. The Student t-test was used to test significant differences between groups. For sphingolipid analysis, groups of siblings (30 per each group) were used for lipid extraction. For the qPCR studies, groups of siblings (minimum 10 per each group) were used to generate cDNAs, and three sets of qPCR were analyzed per each target gene. P values less than 0.05 were considered statistically significant.

## Supplementary information

Supporting data

## Data Availability

The datasets generated or analyzed during this study are included in this published article (and its Supplementary information files).
