# Author Correction: *3-ketodihydrosphingosine reductase* mutation induces steatosis and hepatic injury in zebrafish

**DOI:** 10.1038/s41598-020-67912-8

**Published:** 2020-07-15

**Authors:** Ki-Hoon Park, Zhi-wei Ye, Jie Zhang, Samar M. Hammad, Danyelle M. Townsend, Don C. Rockey, Seok-Hyung Kim

**Affiliations:** 1 Department of Medicine, Medical University of South Carolina, Charleston, SC 29425 USA; 2 Department of Cell and Molecular Pharmacology and Experimental Therapeutics, Medical University of South Carolina, Charleston, SC 29425 USA; 3 Department of Regenerative Medicine and Cell Biology, Medical University of South Carolina, Charleston, SC 29425 USA

Correction to: *Scientific Reports* 10.1038/s41598-018-37946-0, published online 04 February 2019

This Article contains an error in Figure 8 where part E is missing. The correct Figure 8 appears below as Figure 1.

**Figure 1:**
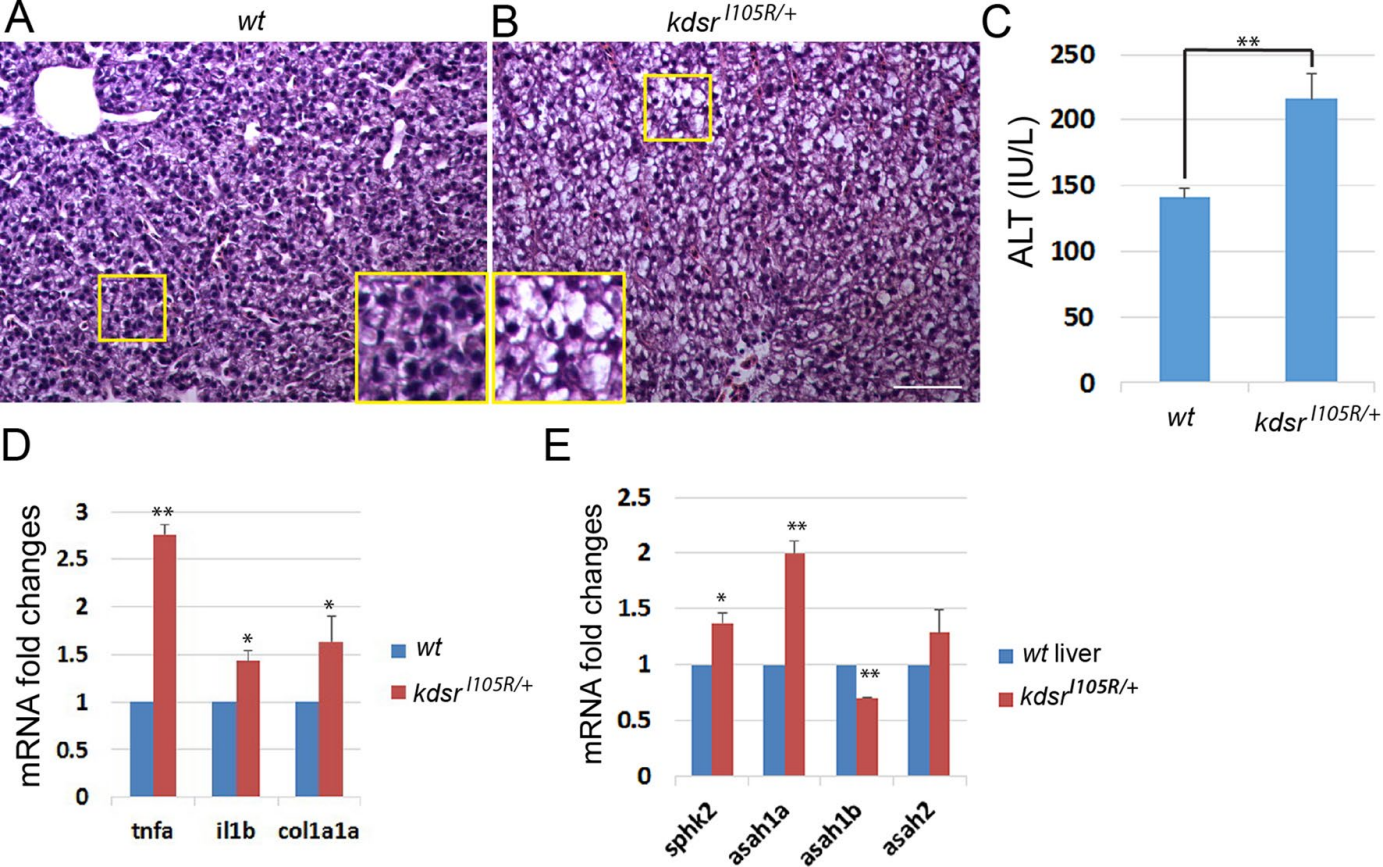
Predisposed liver injury in the *kdsr*^*I105R*/+^ adult zebrafish. H & E staining of wild type liver (**A**) and heterozygous *kdsr* mutant liver (**B**). The inset depicts high resolution of the identified areas. Images shown are representative of at least 10 in total. Scale bar for A and B is 100 μm. Serum ALT test (**C**) in wild type and heterozygous kdsr mutant (n = 3). Relative mRNA expression of *tnfa*, *il1b*, and *col1a1a* (**D**) and *sphk2*, *asah1a*, *asah1b*, and *asah2* (**E**) in wild type and *kdsr*^*I105R*/+^ mutants (n = 3). Error bars indicate standard deviation of the mean. *P ≤ 0.05, **P ≤ 0.005. ALT, alanine aminotransferas.

