# Supplementary material for: 3-ketodihydrosphingosine reductase mutation induces steatosis and hepatic injury in zebrafish
Source: Sci Rep. 2019 Feb 4;9:1138. doi: 10.1038/s41598-018-37946-0 (PMC6361991; doi:10.1038/s41598-018-37946-0)
Supplement: Supplementary file 1 — Supporting data [file 41598_2018_37946_MOESM1_ESM.pdf]

Supplemental information:

***3-ketodihydrosphingosine reductase* mutation induces steatosis and hepatic injury in zebrafish**

Ki-Hoon Park[1], Zhi-wei Ye[2], Jie Zhang[2], Samar M. Hammad[3], Danyelle M. Townsend[2], Don C. Rockey[1], Seok-Hyung Kim[1, 3,*]

[1] Department of Medicine, Medical University of South Carolina, Charleston, SC, 29425, USA.

[2] Department of Cell and Molecular Pharmacology and Experimental Therapeutics, Medical University of South Carolina, Charleston, SC, 29425, USA.

[3] Department of Regenerative Medicine and Cell Biology, Medical University of South Carolina, Charleston, SC, 29425, USA.

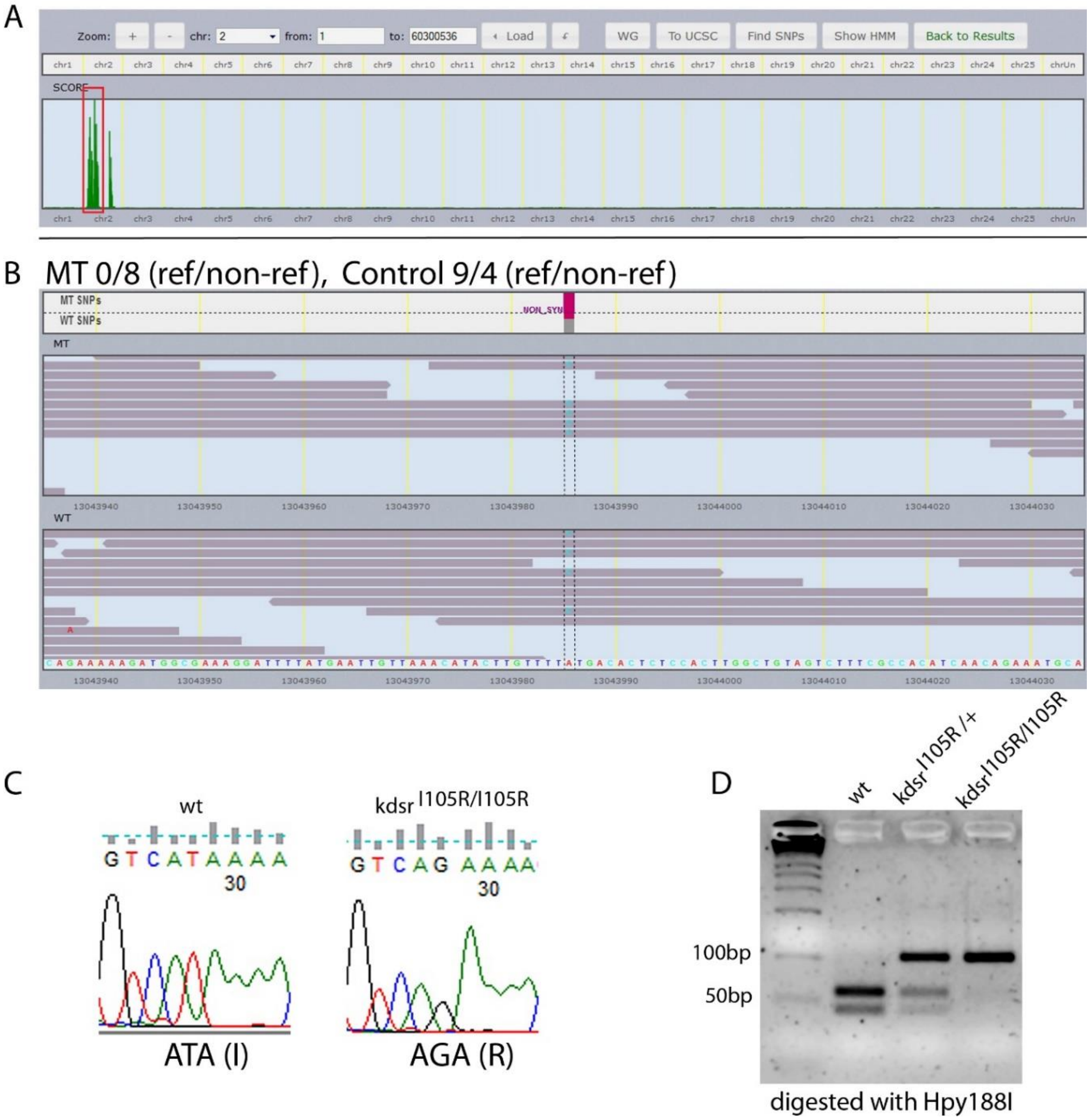

Supporting Fig. 1. SNPTrack analysis and sequencing of heterozygous and homozygous *kdsr*^{I105R} mutant. In (A) is depicted a distribution of SNPs in whole genome, implying that the highest probability of linked mutation causing the mutant phenotype. (B) Homozygous mutant pool carries A(T)->C(G) conversion in kdsr. 8 reads (C, muted sequence) vs 0 read (A, reference sequence) in homozygous mutant and 9 reads (C, mutated sequence) and 4 reads (A, reference sequence) found in normal looking control siblings. (C) Confirmation of the T to G mutation in *kdsr* from cDNAs of wild type and homozygous *kdsr* mutant larvae. (D) Genotyping image of wild type, *kdsr*$^{I105R/+}$ and *kdsr*$^{I105R/I105R}$ mutants. A 2% agarose gel was used for electrophoresis and PCR fragments from genomic DNAs were digested with HpyI188I, which cuts wild type sequence.

Note. We found another SNP causing D39 to H in finTRIM family, member 1 (fintrim01) that is closely linked to *kdsr* locus. finTRIMs are a large new subfamily of TRIMs in teloests and zebrafish and has 84

genes may have functional redundancy *. In mammals, TRIM family members are involved in innate immunity against viral infection. The amino acid residue was not highly conserved in other vertebrates including mice and human. Thus, the additional missense mutation in fintrim01 may not affect the mutant phenotype. To validate the role of kdsr in zebrafish, we generated a *kdsr* knockout model using CRISPR/Cas9 gene modification approach (see supporting figure 3 and 4).

* Van der Aa, L. M. et al. A large new subset of TRIM genes highly diversified by duplication and positive selection in teleost fish. BMC Biol 7, 7, doi:10.1186/1741-7007-7-7 (2009).

```
Identities; 270/332 (81%), Positives; 310/332 (93%)

zkdsr  1    MLLVVAAFIVAFVLLLYMISPLISPKPLKLNGAHVVVTGGSSGIGKCIAMECYKHGAFIT  60
            MLL+ AAF+VAFVLLLYM+SPLISPKPL L GAHVVVTGGSSGIGKCIA+ECYK GAFIT
hKDSR  1    MLLLAAAFLVAFVLLLYMVSPLISPKPLALPGAHVVVTGGSSGIGKCIAIECYKQGAFIT  60

zkdsr  61   LVARDEHKLVQAKKEVEKFAINDKQVVLCISVDVAKDYSQVESVIKQAQEKLGPVDMLVN  120
            LVAR+E KL+QAKKE+E  +INDKQVVLCISVDV++DY+QVE+VIKQAQEKLGPVDMLVN
hKDSR  61   LVARNEDKLLQAKKEIEMHSINDKQVVLCISVDVSQDYNQVENVIKQAQEKLGPVDMLVN  120

zkdsr  121  CAGTSLSGKFEEVEVDHFKKMMEVNYLGSVYPTRAVITTMKERRMGRIMFVSSQAGQIGL  180
            CAG ++SGKFE++EV  F+++M +NYLGSVYP+RAVITTMKERR+GRI+FVSSQAGQ+GL
hKDSR  121  CAGMAVSGKFEDLEVSTFERLMSINYLGSVYPSRAVITTMKERRVGRIVFVSSQAGQLGL  180

zkdsr  181  FGYTAYSPSKFALRGLAEALQMEMKPYNIYVTVAYPPDTDTPGFAEENKTKPLETKLISE  240
            FG+TAYS SKFA+RGLAEALQME+KPYN+Y+TVAYPPDTDTPGFAEEN+TKPLET+LISE
hKDSR  181  FGFTAYSASKFAIRGLAEALQMEVKPYNVYITVAYPPDTDTPGFAEENRTKPLETRLISE  240

zkdsr  241  TSGVSQPEQVAKIVVKDAVQGNFTSSFGPDGYMLSALTCGMSPVTSITEGLQQIVTMGLF  300
            T+ V +PEQVAK +VKDA+QGNF SS G DGYMLSALTCGM+PVTSITEGLQQ+VTMGLF
hKDSR  241  TTSVCKPEQVAKQIVKDAIQGNFNSSLGSDGYMLSALTCGMAPVTSITEGLQQVVTMGLF  300

zkdsr  301  RTIALFYLGSFDSIVRRCMIQREQCKAADKRE  332
            RTIALFYLGSFDSIVRRCM+QRE+ + ADK
hKDSR  301  RTIALFYLGSFDSIVRRCMMQREKSENADKTA  332
```

Supporting Fig. 2. Protein homology comparison of human KDSR and zebrafish kdsr. The red colored amino acid residue indicates mutated amino acid in zebrafish *kdsr*^{I105R} mutant.

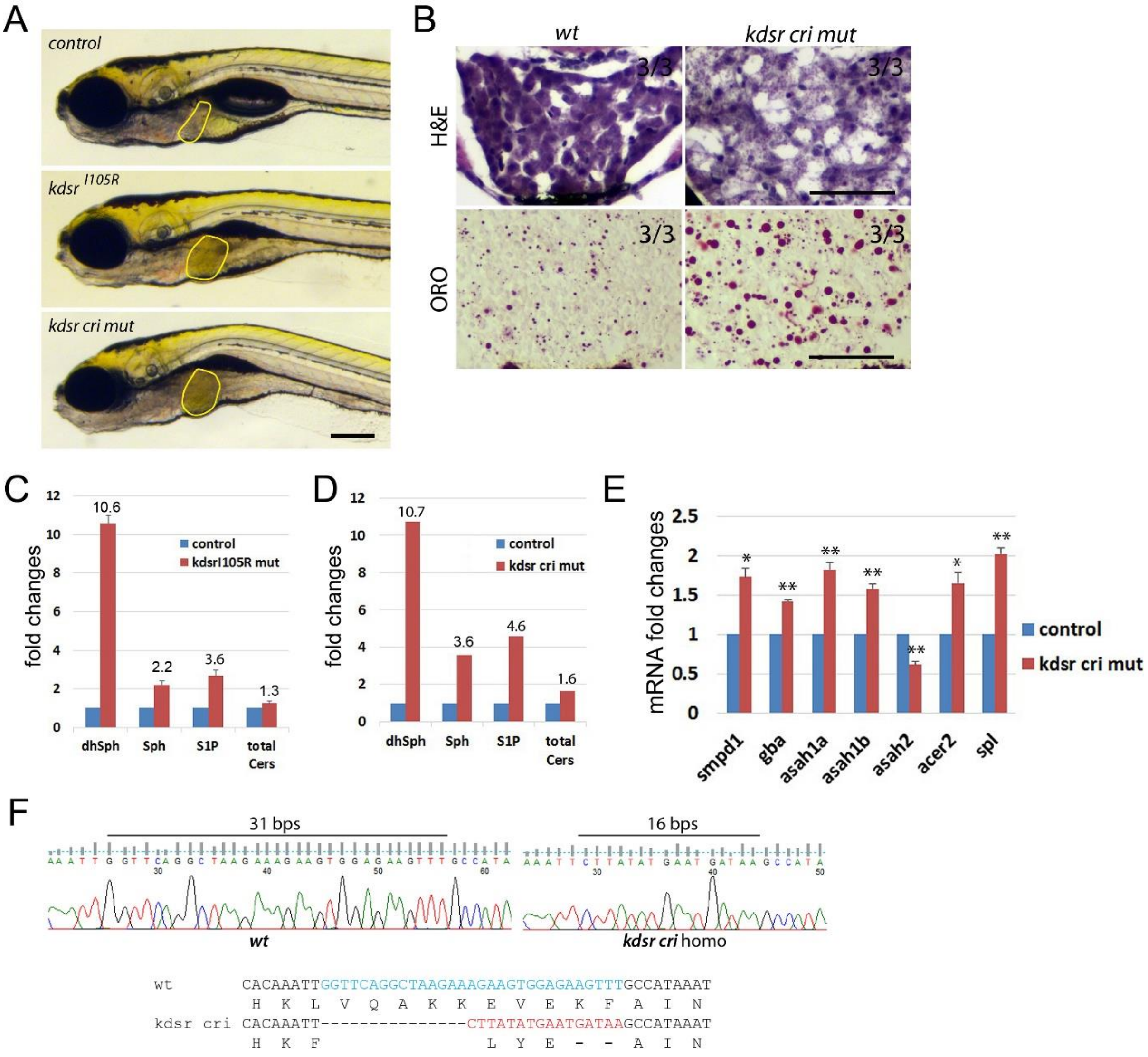

Supporting Fig. 3. The *kdsr*^{cri} mutant generated by CRISPR gene targeting and sphingolipids profiling. Wild type and the *kdsr*^{cri} mutant images at 7 dpf (A), Scale bar=0.25 mm. H & E staining and ORO staining in control (B, left), mutant at 7 dpf (B, right). Scale bar=50µm. Relative amount of sphingolipids from 30 control siblings and 30 *kdsr*^{I105R} mutant larvae siblings at 7 dpf (C) and 30 control siblings and 30 *kdsr*^{cri} mutant larvae siblings at 7 dpf (D). Relative mRNA expression in sphingolipid salvage pathway components in control siblings and *kdsr*^{cri} mutants at 7 dpf (E). CRISPR/Cas9 RNA injection generated premature stop codon in exon3 of *kdsr* in the *kdsr*^{cri} mutant by 31 bps deletion and 16 bps insertion (F). * P≤0.05, ** P≤0.005.

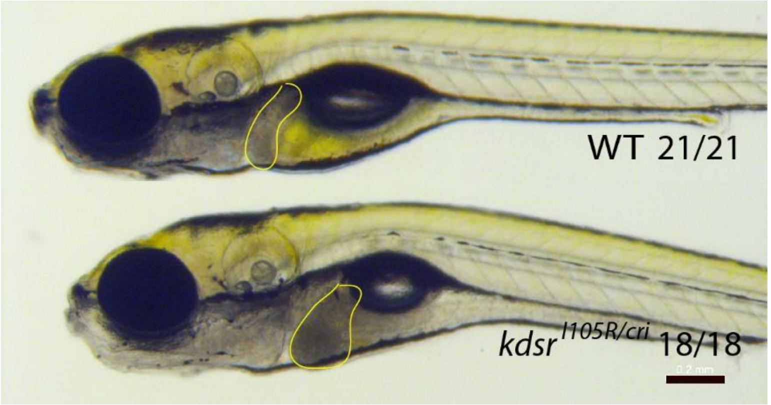

Supporting Fig. 4. *kdsr*$^{I105R/cri}$ biallelic mutants showed hepatomegaly and darker liver at 8 dpf. Liver is outlined with yellow line. Scale bar=0.2mm.

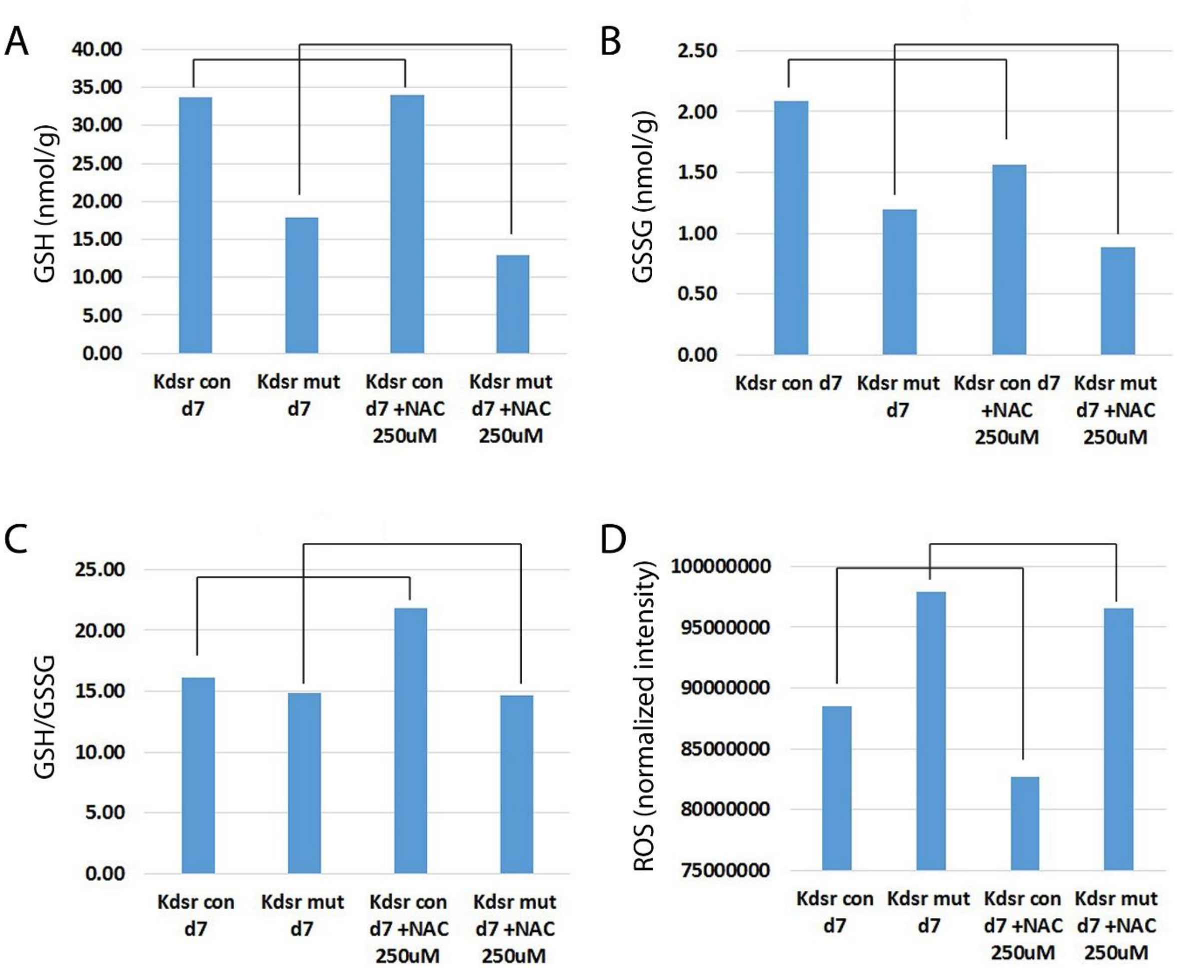

Supporting Fig. 5. N-acetyl cysteine (NAC) treatment in kdsr control and mutant larvae. NAC treatment did not increase GSH synthesis (A). Decrease of oxidized GSH (GSSG) by NAC treatment (B) resulted in increase of GSH/GSSG ratio in the control siblings (C). ROS level was decreased in control by NAC treatment but NAC did not suppress elevated ROS in the *kdsr*^{I105R} mutant larvae (D). 250uM NAC was treated in control and mutant larvae from 4 to 7 dpf. 3 larvae per group were used for analysis.

Note. ROS was measured by using OxiSelect In Vitro ROS/RNS Kit from Cell Biolabs (San Diego, CA), as per the manufacturer's protocol. The fluorescent intensity of fluorophore dichlorofluorescein was detected at 480Ex/530Em by SpectraMax M5 Multi-Mode Microplate Reader (Molecular Devices, Sunnyvale, CA).

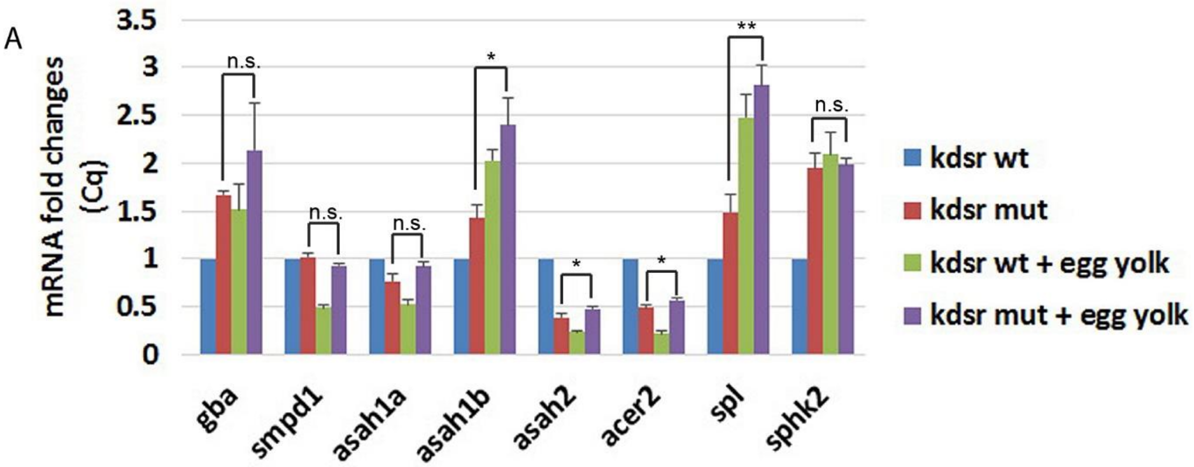

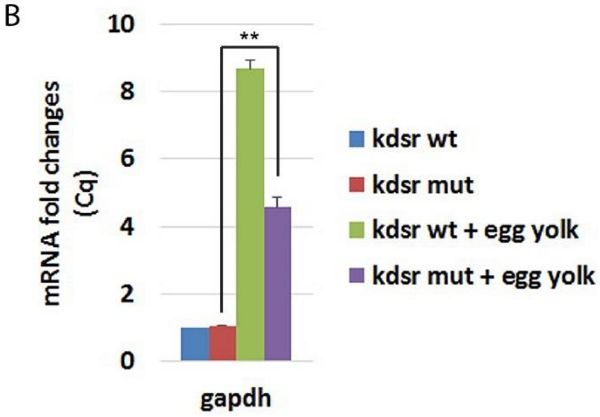

Supporting Fig. 6. External nutrition induced genes involved in the sphingolipid salvage pathway. 6 days-old wild type control and *kdsr*^{I105R} mutant siblings were incubated in 5% chicken egg yolk solution for 24 hours. Larvae consumed egg yolk were collected and piece of tail was used for genotyping. Wild type and homozygous mutant larvae were used for RNA extraction (n=15 per each). (A) mRNA fold changes in genes involved in sphingolipid salvage pathway and sphingosine 1 phosphate metabolism. We used Cq value in wild type without feeding as control, because feeding larvae altered expression of a reference gene (*gapdh*) in both wild type and mutant larvae (B). * P≤0.05, ** P≤0.005.

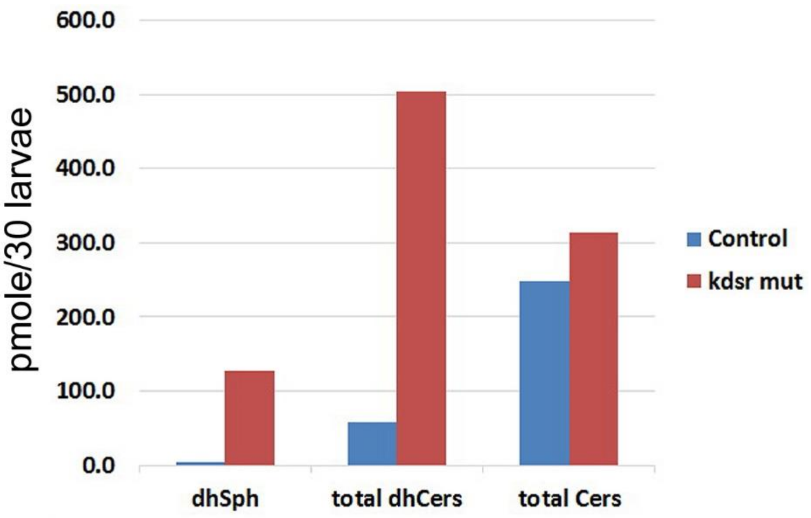

Supporting Fig. 7. Accumulation of dihydro-Sph, total dihydro-Ceramides and Ceramides by loss of kdsr function. The representative graphs of lipid analysis was obtained from each 30 larvae of control siblings and mutant.

symbol	*gene name*		primer sequence	Reference
tnfa	*tumor necrosis factor alpha*	F<br>R	CAGGGCAATCAACAAGATGG<br>TGGTCCTGGTCATCTCTCCA	49
il1b	*interleukin 1 beta*	F<br>R	GGCTGTGTGTTTGGGAATCT<br>TGATAAACCAACCGGGACA	49
a1at	*alpha-1 antitrypsin*	F<br>R	CATGTTGGGTCACAGTCAGG<br>CGATTTCAGGCTTGGAGAA	453
col1a1a	*collagen type 1 alpha 1a*	F<br>R	GCTTTTGGCAAGAGGACAAG<br>TGTCTTCGCAGATCACTTCG	48
srebp1	*sterol regulatory element-binding protein 1*	F<br>R	ACTCTTCTGGTGTGGCTGCT<br>GAGCCTTCAGACACGTCCTC	53
fasn	*fatty acid synthase*	F<br>R	ACACGGTTCACGCATTTGTG<br>GACCCATCTTCCGTAGCATATCA	48
srebp2	*sterol regulatory element-binding protein 2*	F<br>R	CACTCACACAAGCACACACG<br>ACCTGGTTCTGGATGAATCG	53
lpl	*lipoprotein lipase*	F<br>R	CTGAGGGCTCTCGTTCATAAAGA<br>AATCCATCAAAGACTGTAACTTCAATACA	48
cpt1	*carnitine-palmitoyltransferase I*	F<br>R	CATCCTTAGGCCTGCTCTTCAAA<br>ACCATGACACCCCCAACTAACAT	
drp1	*dynamin related protein 1*	F<br>R	AGCCAGTCAGGTGATCGCCGA<br>CGCAGGGTTCGCGTGAAGGG	52
mfn1	*mitofusin 1*	F<br>R	CTGGGTCCCGTCAACGCCAA<br>ACTGAACCACCGCTGGGGCT	52
opa1	*optic atrophy type 1*	F<br>R	GCCGGAAGTGTAGTTACCTG<br>AGGTGGTCTCTGTGGGTTGT	52
pgc1a	*peroxisome proliferator-activated receptor gamma coactivator 1-alpha*	F<br>R	GGCCCAGCGAGCCAAACCAA<br>TGGCTTTGTGAGGAGGCGTGG	52
nd1	*NADH-ubiquinone oxidoreductase chain1*	F<br>R	GGGCACCCATACCCATGCCCTAT<br>TGCGCTACAGCTCGTAAGGC	52
smpd1	*sphingomyelin phosphodiesterase 1*	F<br>R	GGTACCTCTTTCACAAAGGCCACGT<br>TCAGCACATGCTCTTCTTGGGTGTG	
asah1a	*acid ceramidase 1a*	F<br>R	GCTTGGAAGATGGTACGTCTTGGAG<br>CGCTTGTGTGGTTTGATTCATGCAC	
asah1b	*acid ceramidase 1b*	F	GGATTCTGGAGTGGATCTTGGGAAAGA	

		R	TATCAGACAGCAGAGCCTTGGCACT	
asah2	*neutral ceramidase 2*	F<br>R	GTTTCACTGGATTAAAGGACTCGCGG<br>CCACGTATGGTGTGATGACTGTGGT	
acer2	*alkaline ceramidase 2*	F<br>R	CACATCCTCATCTGTCTGGCCTCA<br>CGATGAAAGCCCACTTCTCACTGG	
spl	*sphingosine 1 phosphate lyase*	F<br>R	TCGGGGTAGTATGGCTAAAGG<br>TCCAGAGCTTTGTTGAGC	51
sphk1	*sphingosine kinase 1*	F<br>R	TGGAGACGGGCTACTCTTTG<br>GTAATGATGGACGGAAGCAG	51
sphk2	*sphingosine kinase 2*	F<br>R	AATTCTGCCTTGTGGCTCTG<br>GCAGAAAACAGCAGTTGAGG	51
spp1	*S1P-phosphohydrolase 1*	F<br>R	ATACCCCTGAGCCTGTTTCTG<br>TAGATGCGGCTCAAACACAC	51
nrf2a	*nuclear factor erythroid 2 -related factor 2*	F<br>R	ACCCAATAGATCTACAGAGC<br>GGTGTTTGGACATCATCTCG	48
sod2	*superoxide dismutase 2*	F<br>R	AGCGTGACTTTGGCTCATTT<br>ATGAGACCTGTGGTCCCTTG	53
gstp1/2	*glutathione S transferase pi 1/2*	F<br>R	CTACAACCTGTTCGATCTCCT<br>GGGCAGAGATCTTGTCCAC	48
gpx1a	*glutathione peroxidase 1a*	F<br>R	GTAAACCAGCGGCTTCTACG<br>GGCACTTTAATCATGACTGCAC	53
gpx4a	*glutathione peroxidase 4a*	F<br>R	CAGGAACCAGGAACTAATTCCCAAATC<br>TCCAAGGAATCCTTTTCCATTAGGCT	
prdx4	*peroxiredoxin 4*	F<br>R	CACGTTTGTGTGTCCGACTGAGAT<br>CAGGCCAGATGAGTAAACTGAGAGT	
txnl1	*thioredoxin-like 1*	F<br>R	TCACAATGGCCTTCAATCAA<br>CCAGACTCTGTGTGGCTTCA	53
txnl4a	*thioredoxin-like 4a*	F<br>R	CACAATGGCTGGCAAGTAGA<br>CTGGCACTTCTGTGATGTCC	53
atf4	*activating transcription factor 4*	F<br>R	TTAGCGATTGCTCCGATAGC<br>GCTGCGGTTTTATTCTGCTC	54
gadd45a	*growth arrest and DNA-damage-inducible 45 alpha*	F<br>R	TCTCATCCAGGCTTTCTGCT<br>GCAGAAGCGGTTCACTTTTC	46
ire1	*inositol-requiring enzyme 1*	F<br>R	CCACAGACTTCATCCACACAGCAC<br>GGCACTATGGAGCTCCAGTCTTCTC	
nfkb	*nuclear factor kappa-light-*	F	CTGCTCTGCGACAAAGTCCAGAAAG	

	chain-enhancer of activated B cells 1	R	AGGAGTCTTGAAGACGATGGCAAAC	
xbp1-u	*X-box binding protein 1-unspliced*	F<br>R	GGGTTGGATACCTTGGAAA<br>AGGGCCAGGGCTGTGAGTA	54
xbp1-s	*X-box binding protein 1-spliced*	F<br>R	TGTTGCGAGACAAGACGA<br>CCTGCACCTGCTGCGGACT	54
atf6	*activating transcription factor 6*	F<br>R	CTGTGGTGAAACCTCCACCT<br>CATGGTGACCACAGGAGATG	54
ddit3	*DNA damage-inducible transcript 3*	F<br>R	AAGGAAAGTGCAGGAGCTGA<br>TCACGCTCTCCACAAGAAGA	54
edem1	*ER degradation-enhancing alpha-mannosidase-like 1*	F<br>R	ATCCAAAGAAGATCGCATGG<br>TCTCTCCCTGAAACGCTGAT	54
bip	*binding immunoglobulin protein*	F<br>R	ATCAGATCTGGCCAAAATGC<br>CCACGTATGACGGAGTGATG	53
dnajc3	*dnaJ homolog subfamily C member 3*	F<br>R	TCCCATGGATCCTGAGAGTC<br>CTCCTGTGTGTGAGGGGTCT	53
grp94	*glucose-regulated protein 94*	F<br>R	AGCAAGACCGAGACCGTAGA<br>CTCCCAATCCCACACAGTCT	53
bim	*bcl-2 Interacting Mediator Of Cell Death*	F<br>R	GAGGGATATCCATGTCGAATAACCAGTCG<br>CATCATTTTGTCCGTCTTGCGCTTCG	
bida	*bh3-interacting domain death agonist a*	F<br>R	GAGAAATGGCGGCAGAGTTGATCAG<br>CCAAACCTGTTCTTGGGAAGATCTCAG	
baxb	*bcl2 associated x b*	F<br>R	ATGAACAGTATCCCTGCACTGGCC<br>CTGAAATCTCGAGACGATGAAGCCACC	
gss	*glutathione synthase*	F<br>R	CAGTGAACTGGGTGCATTCGGG<br>GTGGATTGTCCAGCACTGCTACTC	
gsr	*glutathione reductase*	F<br>R	GTGGTCGGTCTCCACATGCAG<br>CGTTGGGTGGATGGCAATGGTTC	
gclc	*glutamate-cysteine ligase, catalytic subunit*	F<br>R	GTGATCTGATGACCATGGCCAAGTG<br>TCTGTCACACTTGTGGAGCAGGTC	
gapdh	*glyceraldehyde-3-phosphate dehydrogenase*	F<br>R	GTGGAGTCTACTGGTGTCTTC<br>GTGCAGGAGGCATTGCTTACA	50

Supporting Table 1. Primer sequences used for qPCRs.

“Primers for highlighted genes with yellow are designed and tested in Dr. Kim’s lab."

47 Cinaroglu A, Gao C, Imrie D, Sadler KC. Activating transcription factor 6 plays protective and pathological roles in steatosis due to endoplasmic reticulum stress in zebrafish. Hepatology 2011;54:495-508

48 Landgraf K, Schuster S, Meusel A, Garten A, Riemer T, Schleinitz D, Kiess W, et al. Short-term overfeeding of zebrafish with normal or high-fat diet as a model for the development of metabolically healthy versus unhealthy obesity. BMC Physiol 2017;17:4

49 Marjoram L, Alvers A, Deerhake ME, Bagwell J, Mankiewicz J, Cocchiaro JL, Beerman RW, et al. Epigenetic control of intestinal barrier function and inflammation in zebrafish. Proc Natl Acad Sci U S A 2015;112:2770-2775

50 McCurley AT, Callard GV. Characterization of housekeeping genes in zebrafish: male-female differences and effects of tissue type, developmental stage and chemical treatment. BMC Mol Biol 2008;9:102

51 Mendelson K, Zygmunt T, Torres-Vazquez J, Evans T, Hla T. Sphingosine 1-phosphate receptor signaling regulates proper embryonic vascular patterning. J Biol Chem 2013;288:2143-2156

52 Rahn JJ, Stackley KD, Chan SS. Opa1 is required for proper mitochondrial metabolism in early development. PLoS One 2013;8:e59218

53 Tsedensodnom O, Vacaru AM, Howarth DL, Yin C, Sadler KC. Ethanol metabolism and oxidative stress are required for unfolded protein response activation and steatosis in zebrafish with alcoholic liver disease. Dis Model Mech 2013;6:1213-1226

54 Vacaru AM, Di Narzo AF, Howarth DL, Tsedensodnom O, Imrie D, Cinaroglu A, Amin S, et al. Molecularly defined unfolded protein response subclasses have distinct correlations with fatty liver disease in zebrafish. Dis Model Mech 2014;7:823-835
